# Quasi-HKUST Prepared via Postsynthetic Defect Engineering for Highly Improved Catalytic Conversion of 4-Nitrophenol

**DOI:** 10.1021/acsami.1c19862

**Published:** 2021-12-31

**Authors:** Minoo Bagheri, Arianna Melillo, Belen Ferrer, Mohammad Yaser Masoomi, Hermenegildo Garcia

**Affiliations:** † Department of Chemistry, Faculty of Science, Arak University, Arak 3848177584, Iran; ‡ Instituto Universitario de Tecnología Química Consejo Superior de Investigaciones Científica and Departamento de Química, Universitat Politecnica de Valencia, Av. De los Naranjos s/n, Valencia 46022, Spain

**Keywords:** heterogeneous catalysis, metal−organic frameworks, defect engineering, partial ligand removal, 4-nitrophenol reduction

## Abstract

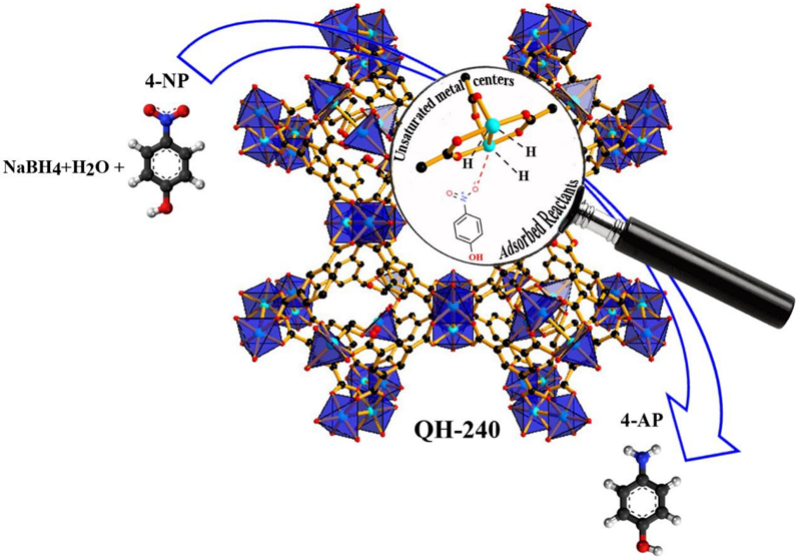

HKUST-1 [Cu_3_(BTC)_2_(H_2_O)_3_]_*n*_·*n*H_2_OMeOH was submitted to thermolysis under controlled conditions at temperatures between 100 and 300 °C. This treatment resulted in partial ligand decarboxylation, generating coordinatively unsaturated Cu^2+^ sites with extra porosity on the way to the transformation of the initial HKUST-1 framework to CuO. The obtained materials retaining in part the HKUST-1 original crystal structure (quasi-MOFs) were used to promote 4-nitrophenol conversion to 4-aminophenol. Because of the partial linker decomposition, the quasi-MOF treated at 240 °C contains coordinatively unsaturated Cu^2+^ ions distributed throughout the Q-HKUST lattice together with micro- and mesopores. These defects explain the excellent catalytic performance of QH-240 with an apparent rate constant of 1.02 × 10^–2^ s^–1^ in excess of NaBH_4_ and an activity factor and half-life time of 51 s^–1^g^–1^ and 68 s, respectively, which is much better than that of the HKUST parent. Also, the induction period decreases from the order of minutes to seconds in the presence of the HKUST and QH-240 catalysts, respectively. Kinetic studies fit with the Langmuir–Hinshelwood theory in which both 4-nitrophenol and BH_4_^–^ should be adsorbed onto the catalyst surface. The values of the true rate constant (*k*), the adsorption constants of 4-nitrophenol and BH_4_^–^ (*K*_4-NP_ and *K*_BH_4_^–^_), as well as the activation energy are in agreement with a rate-determining step involving the reduction of 4-nitrophenol by the surface-bound hydrogen species.

## Introduction

As it has been widely recognized, water pollution is a worldwide problem that greatly impacts living ecosystems. Nowadays, much research has been focused on the removal of water contaminants, such as aromatic pollutants.^1^ 4-Nitrophenol (4-NP) is a well-known toxic compound with high solubility and stability in water. It is a widely used synthetic intermediate in chemical processes to produce drugs, dyes, explosives, and pesticides. However, due to its biotoxicity, it is difficult to decompose by natural microbial degradation. Thus, the development of a cost-effective and ecofriendly method to remove this compound especially from agricultural and industrial wastewater, is of great importance.^2^

The chemical reduction of 4-NP to 4-aminophenol (4-AP) can be a convenient strategy to remediate 4-NP wastes, since 4-AP is an important intermediate for the synthesis of analgesics and drugs, photographic developers, corrosion inhibitors, anticorrosion lubricants, and other specialty chemicals.^3^

Several approaches have been applied for the reduction of 4-NP, including electrolytic reduction, metal-acid reduction, and catalytic hydrogenation. Electrolytic and metal-acid reduction reactions suffer from some disadvantages such as the need of an acidic or alkaline electrolyte, low efficiency, and poor selectivity. In comparison, the catalytic hydrogenation can be considered as a good prospective procedure to valorize 4-NP without generating any acidic waste, reaching high conversion and selectivity under mild conditions.^4^ Hence, recent studies have been more concerned with the efficiency of catalytic reduction of 4-NP into less toxic and valuable 4-AP, which is not only important from the environmental remediation point of view, but also beneficial as a valorization process. This catalytic reduction of 4-NP can be performed using sodium borohydride (NaBH_4_), which is a mild reducing reagent, and the reaction can be conducted in aqueous medium under ambient conditions.^5^ Many studies have reported various catalytic systems.^6^ Among them, Cu-based complexes are convenient and efficient as Lewis acid catalysts combined with copper availability, economic affordability, and low toxicity. Therefore, several studies have reported on the catalytic activity of copper compounds and copper oxides as catalysts for 4-NP reduction by NaBH_4_.^7−11^

Metal–organic frameworks (MOFs), consisting of organic linkers and inorganic nodes defining a porous lattice, are among the most versatile heterogeneous catalysts.^12−14^ The active sites of MOFs can be coordinatively unsaturated metal ions, functional groups on the organic ligands, and/or guests inside the pores. Although there is a large number of MOFs with inherent catalytic properties, MOFs can also be used as supports, used as precursors of other materials, or combined with other active components.^15−17^ Two methods are generally used to enhance the activity of MOFs as catalysts: (1) pore engineering to facilitate accessibility to the active sites and intracrystalline diffusion of substrates, reagent, and products,^18^ and (2) the generation of a coordinatively unsaturated position around metal sites by removal of solvent molecules.^19,20^ Pore engineering and structural defects have been generated during the synthesis by means of modulators that compete with the organic ligand during the formation of the coordinative bonds between the metal clusters and the linker. Alternatively, modification of porosity and generation of defects can be achieved by postsynthetic methods. Among them, the controlled thermal partial ligand decomposition has been proposed recently to create Lewis acid sites.^21−23^ The resulting materials are denoted as quasi-MOFs.^23,24^ It must be noted that defective MOFs have vacancies either of linkers or of metal nodes, whereas quasi-MOFs are materials with structures between the initial highly crystalline MOFs and the final metal oxides acquired by complete deligandation at relatively high temperatures.^23,24^ Control of the structural damage caused by heating of the MOF would allow one to prepare a new generation of materials with lower crystallinity, but with enhanced catalytic performance. The exact increase in activity that can be achieved following this strategy is still unknown, and it is of interest to determine which level of enhancement can be achieved before the material is finally transformed into the metal oxide.

The present study reports on the optimal quasi-Cu-MOF (HKUST-1) to catalyze the reduction of 4-NP by NaBH_4_ as the reductant agent. Our strategy is based on thermal defect generation resulting in pore engineering and an extra high density of open metal sites in HKUST-1 with simultaneous distribution of both micro- and mesopores. These defects arise from the partial deligandation of the Q-HKUST network. Experimental evidence supports that controlled thermolysis can create highly active Lewis acid sites throughout the Q-HKUST along with the coexistence of micro- and mesopores. Contrary to the most widely reported use of metal oxides,^25−27^ and to achieve an enhanced catalytic performance in 4-NP hydrogenation, partial deligandation appears to be an efficient and convenient postsynthetic treatment, avoiding the massive CuO particle aggregation occurring in the complete decomposition of the 1,3,5-benzenetricarboxylate (BTC) ligand in HKUST-1. In this intermediate material, the porosity and skeleton structure of ancient HKUST is still acceptably intact, ensuring the reactant access to the active sites as well as enabling the diffusion of reagents and products.

Up until now, some Q-MOFs have been considered as catalysts in CO oxidation by promoting the formation of active oxygen species,^21,28,29^ the oxidation of benzyl alcohol,^30^ and CO_2_ adsorption.^31^ Numerous studies have been carried out on the development of catalysts for the reduction of 4-NP, including Cu catalysts.^32−37^ However, as far as we know, this is the first report on the employ of quasi-MOFs in the catalytic reduction of 4-NP under moderate conditions.

The present study shows how the controlled partial thermolysis of HKUST-1 can produce the quasi-HKUST that exhibits a much enhanced catalytic activity for 4-NP reduction, as a consequence of the presence of unsaturated Lewis acid sites and the beneficial presence of micro- and mesopores. Q-HKUST has been prepared via partial deligandation in air at different temperatures in the range of 200–300 °C, with the highest catalytic performance for deligandation observed at 240 °C. This Q-HKUST is far more active than the parent pristine HKUST-1. The presence of residual phenyl rings of BTC in the mesopores facilitates 4-NP uptake via π–π stacking and hence increases the catalytic efficiency. Q-HKUST reusability and kinetics were also investigated.

## Experimental Section

### Synthesis of HKUST-1

In a typical experiment, HKUST was obtained by mixing BTC (2.1 g, 10 mmol) and Cu(OAc)_2_·H_2_O (3.21 g, 16 mmol) in 400 mL of H_2_O:EtOH (1:1) in a round-bottom flask at 110 °C for 4 h (see the Supporting Information for more information).

### Synthesis of QH-*x*

The thermal treatment of HKUST-1 was performed in air at temperatures between 100 and 400 °C with heating at a 5 °C min^–1^ rate for a period from 30 min to 2 h. The samples were denoted as QH-*x*, where “H” and “*x*” stand for HKUST and the deligandation temperature, respectively. Figure S1 shows the IR spectra of the resulting QH-*x* samples.

### Catalytic Evaluation

The catalytic reduction of 4-NP was followed by UV–vis absorption spectroscopy. Before each run, the solutions were purged with N_2_ to remove O_2_. For this reason, 1.0 mg of catalyst was added to 25 mL of freshly prepared 4-NP (0.05 mM) and another 25 mL of NaBH_4_ (10 mM) solutions at room temperature (rt). The solutions then were stirred vigorously. The conversion of 4-NP was subsequently determined by measuring the optical density at λ_max_ = 400 nm at periodic intervals. The catalytic 4-NP reduction performance was determined as follows:
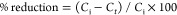
1where *C*_i_ is the initial concentration of 4-NP and *C*_*t*_ is the concentration of 4-NP at any given time.

The kinetics of the reduction reaction can be obtained from eqs 2–4:
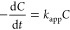
2
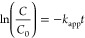
3
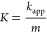
4where *k*_app_ is the apparent reaction rate, *m* is the catalyst mass in gram, and *K* is the activity factor. To evaluate catalyst stability under the reaction conditions, the solid QH-*x* was isolated from the mixture by centrifugation and washed several times with deionized water and ethanol (1:1), and subsequently dried under vacuum at 100 °C for 10 h. The sample then was reused in the next reduction reaction. Four consecutive uses of the same sample were performed following the procedure mentioned above.

## Results and Discussion

**Figure 1:**
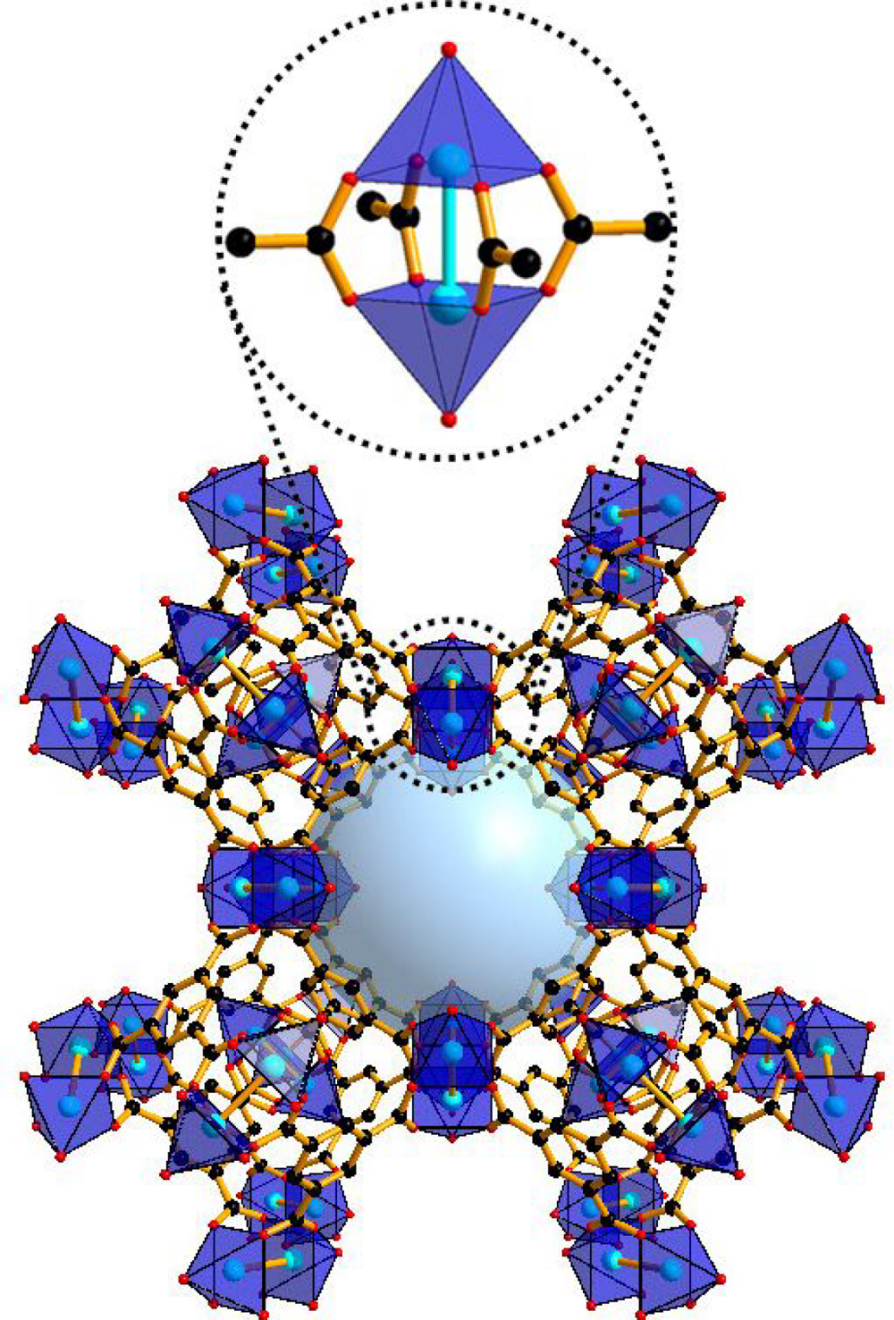
Illustration of the details of the HKUST-1 structure. Color code: O, red; C, black; and Cu, blue.

HKUST-1 was obtained via solvothermal synthesis, by reacting copper(II) acetate and BTC ligand. The structure of HKUST-1 consists of 3D interconnected square-shaped pores (9 Å × 9 Å) fabricated by connecting paddlewheel Cu(II) units through BTC ligands. Each Cu(II) metal ion with an octahedral configuration is coordinated to four different BTC linkers via oxygen atoms plus a Cu–Cu linkage (2.628 Å) and one H_2_O molecule coordinated in the direction of the Cu–Cu axis (Figure 1).

TGA analysis (Figure S2) revealed that thermal decomposition at moderate temperatures should cause partial deligandation of HKUST, generating additional pores and unsaturated inorganic metal centers. For this reason, HKUST-1 was heated at 120 °C for 14 h to expel guest molecules (H_2_O and EtOH) from the pores. Deligandation of the framework was performed by heating at 200, 240, and 260 °C for 30 min and 300 °C for 2 h or 30 min. Evolution of H_2_O and CO_2_ in the process was specified by MS analysis of the gases evolved in the head space upon heating. The corresponding samples are denoted, respectively, as QH-200, QH-240, QH-260, and QH-300.

The morphology of all samples was visualized by FE-SEM. The images revealed that parent HKUST-1 powder contains cubic nanoparticles. Thermal treatment decreases the average particle size, and particle agglomeration was observed (Figures S3–S12). EDS analysis shows the composition of the particles containing C, O, and Cu, while at temperatures of 400 °C only Cu and O can be detected, which indicated the complete conversion of HKUST-1 into CuO/Cu_2_O (Figure S12). In the case of QH-300, the presence of C is barely detectable by EDS (Figure S10).

**Figure 2:**
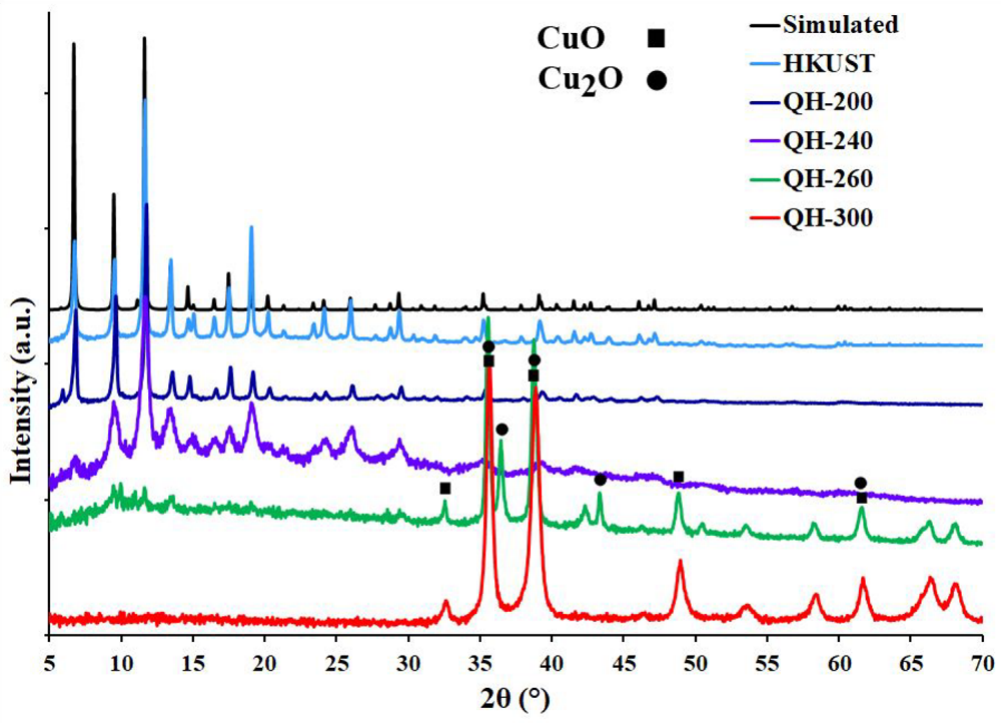
PXRD patterns of the HKUST and QH-*x* samples.

PXRD of HKUST-1 and QH-*x* indicates that the original framework does not undergo significant changes up to 240 °C, except for a slight broadening of the peaks around 5–10° and diminution of their intensity, which indicate the initial stages of structural damage of HKUST. Upon increasing the treatment temperature to 260 °C, the characteristic peaks significantly decrease in intensity, eventually disappearing. For the 260 °C treatment, the presence of peaks corresponding to Cu_2_O/CuO (JCPDS nos. 05-0667 and 48-1548) was observed. When submitted to 300 °C, HKUST-1 is entirely transformed to CuO/Cu_2_O, implying the complete BTC decomposition (Figure 2).

**Figure 3:**
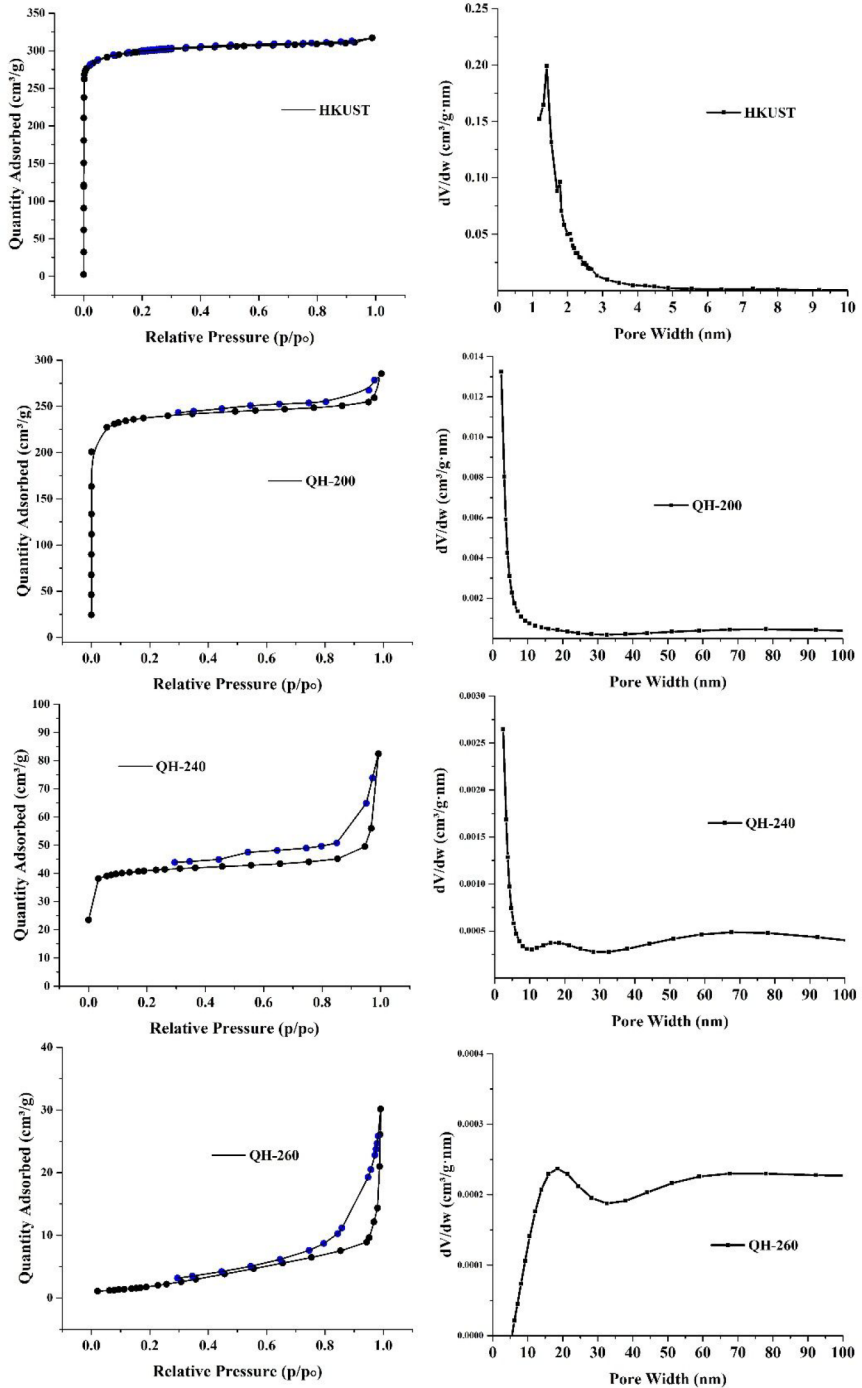
N_2_ isotherm collected at 77 K and 1 bar and pore size distribution for HKUST, QH-200, QH-240, and QH-260.

The N_2_ adsorption data at 77 K for HKUST-1, QH-200, QH-240, and QH-260 display types I, (I, IV), IV, and V isotherms with BET specific surface areas of 1182, 913, 160, and 5.6 m^2^ g^–1^, respectively (Figure 3 and Table S1). Noticeable hysteresis in the adsorption of thermal decomposed samples implies the presence of large cavities. Moreover, the pore width distribution in QH-240 reveals a significant enhancement of the mesopores upon deligandation. The existence of micro- and mesopores in QH-240 can be ascribed to the formation of defects or vacant sites by partial deligandation with CO_2_ evolution and the development of a quasi-MOF structure. As a consequence of deligandation, a uniform distribution of coordinatively unsaturated Cu(II) ions in both micro- and mesopores should be present, and they can contribute to the improvement of the catalytic performance.

**Figure 4:**
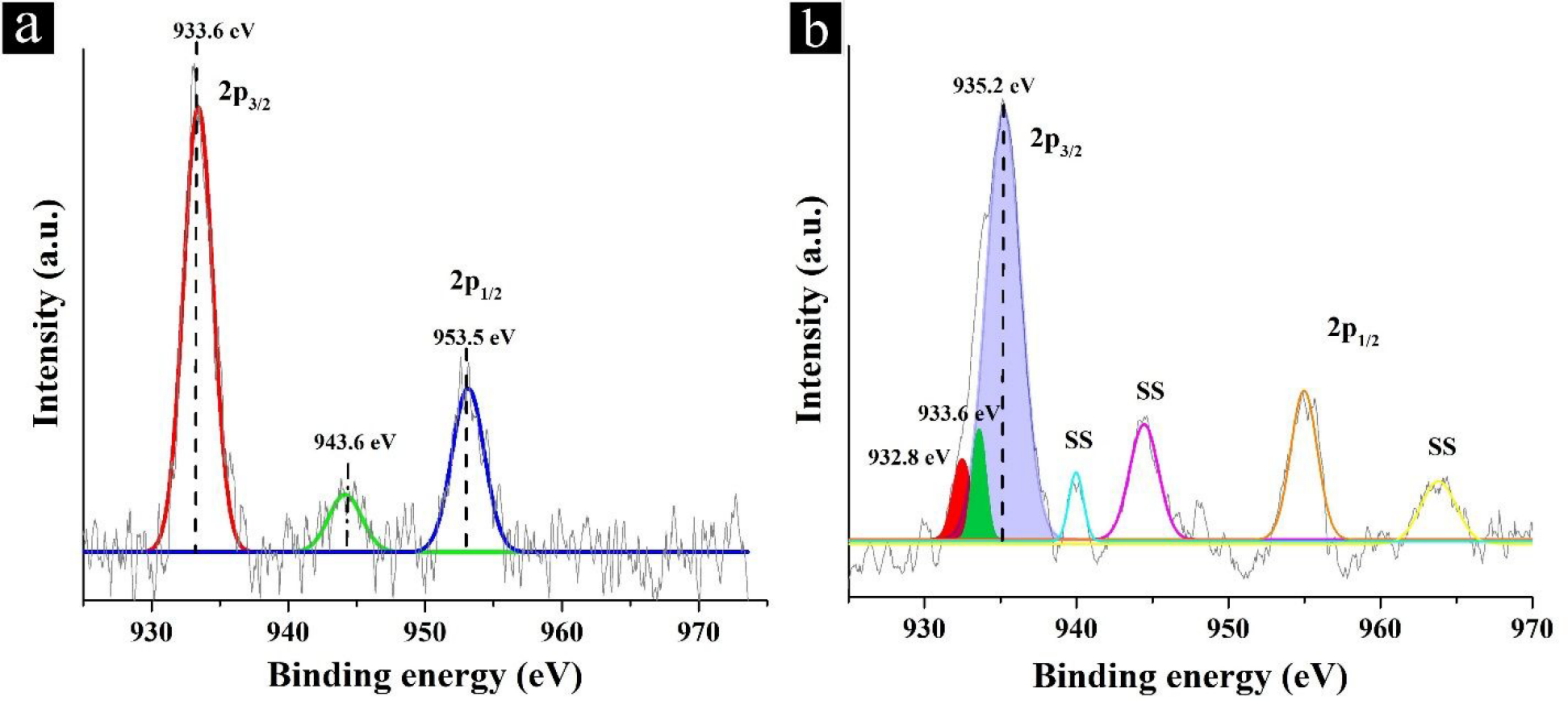
XP spectra of the Cu 2p peaks for the (a) HKUST-1 and (b) QH-240 samples and their best deconvolution to individual components (SS = shake-up satellite).

The XPS analysis was performed to gain insight into the distribution of Cu atoms among the various oxidation states after thermal treatment (Figure 4). In the XPS spectrum of HKUST-1, there are two peaks of Cu 2p_3/2_ and Cu 2p_1/2_ located at 933.6 and 953.5 eV, respectively, and the corresponding shakeup satellite of Cu 2p_3/2_ located at about 10 eV higher at 943.6 eV. All of these peaks agree with octahedral Cu^2+^.^38,39^ For QH-240, a new peak appeared at 932.8 eV attributed to the Cu^+^ species, accompanying a new intense peak at 935.2 eV that can be ascribed to the Cu^2+^ in tetrahedral coordination. These changes are associated with a considerable decrease in the peak intensities of octahedral Cu^2+^ (residual 8%).^40^ Thus, the XPS analysis confirms that a small fraction of Cu^+^ (8%) was created in QH-240 after deligandation as well as the presence of Cu^2+^ in tetrahedral sites (84%). In addition, the XP spectrum of QH-240 after reaction is almost similar to that of pristine QH-240. XPS data in combination with temperature-dependent PXRD showing that the final material after calcination at 300 °C is Cu_2_O indicate the chemical reduction from Cu^2+^ to Cu^+^ in the thermal treatment. One possibility that cannot be ruled out at the moment is the reduction of Cu^2+^ to Cu^0^, followed by the subsequent Cu^2+^ and Cu^0^ comproportionation to Cu^+^. The last possibility could be more likely for QH samples obtained at a higher temperature treatment, in which a massive formation of Cu_2_O is observed.

Despite the dramatic changes shown in PXRD and surface area in the thermal treatment, vibrational spectroscopy that mostly reports on the functional groups of the organic ligand reflects minor variations, except for QH-300 and QH-400 for which the bands of the organic ligands have completely disappeared. Regarding the Raman spectra, the same bands, but much broader as determined by the width at half height, were recorded for HKUST-1 and QH-240. Figures S13 and S14 show the IR spectra of the samples under study and a comparison of the Raman spectra of HKUST-1 and QH-240.

The EPR spectrum of HKUST-1 corresponds to Cu^2+^ in octahedral coordination. However, this characteristic EPR signal diminishes considerably in intensity to about a 10% in QH-240. Figure S15 shows the corresponding EPR spectra, thus reflecting the substantial changes occurring to the Cu^2+^ oxidation state and loss of the octahedral coordination in the thermal treatment.

**Figure 5:**
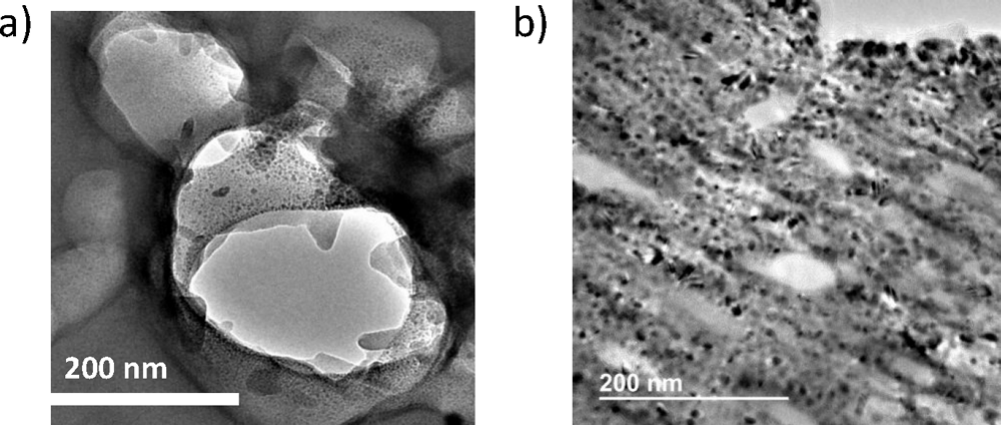
TEM images of (a) HKUST-1 and (b) QH-240. While HKUST shows a clean, smooth surface, QH-260 shows nanoparticles as well as the presence of macropores.

Because of the large particle size, MOFs are not suitable for direct transmission electron microscopy. To observe the changes produced in the deligandation process, HKUST-1 and QH-240 were embedded in a polymeric matrix that allows one to cut thin slices by fast ion bombardment suitable for TEM imaging. The results are presented in Figure 5. As it can be seen there, while no particles are observed in the case of the HKUST-1 image that show a smooth internal surface, the QH-240 image reveals the formation of small CuO/Cu_2_O particles between 3 and 18 nm, with an average of 7 nm. The presence of meso-/macropores in QH-240 can also be observed.

### Catalytic 4-NP Reduction

The catalytic performance of the QH-*x* samples was examined for the reduction of 4-NP to 4-AP by screening some parameters on the catalyst treatment and reaction conditions, such as the deligandation temperature and catalyst mass. Kinetic data of reduction reaction such as activation energy, enthalpy, and entropy values of the adsorption on the most active catalyst were also determined.

By introducing NaBH_4_ to the 4-NP solution, the strong absorption peak in the UV region shifted from 317 to 400 nm, which is associated with the creation of 4-nitrophenolate ions. Preliminary controls indicate that 4-NP reduction does not happen in the absence of catalyst even for a prolonged time. Upon addition of a proper amount of the suitable catalyst and as the reaction progressed, the absorption intensity at 400 nm gradually decreased, while a new peak appeared at 300 nm, indicating the production of 4-AP. Also, with the completion of the reduction reaction, the bright yellow solution became colorless (Figure S16).

### Influence of Deligandation Temperature

**Figure 6:**
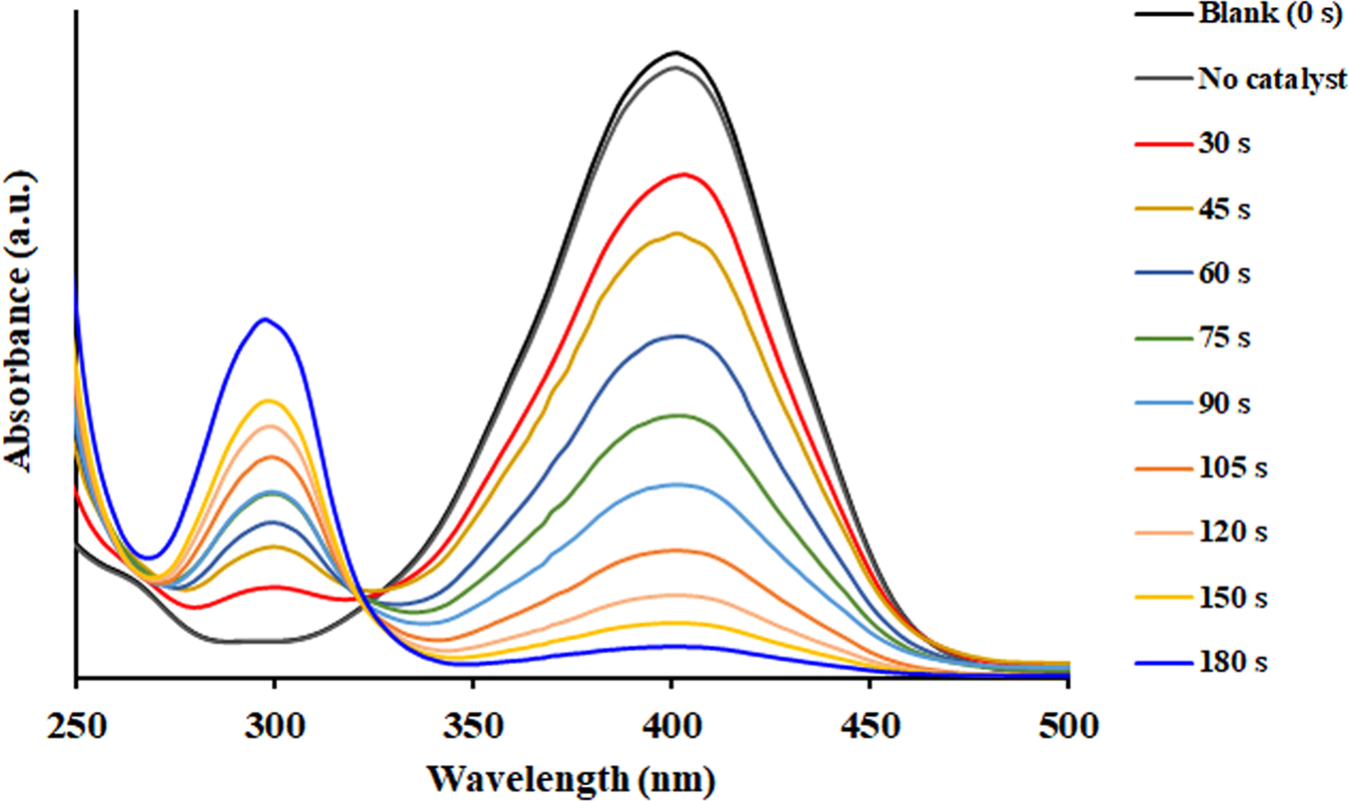
UV–vis spectra of 4-NP and NaBH_4_ solution after 1.0 mg of QH-240 catalyst was added.

The effect of temperature treatment on the catalytic activity of the material for 4-NP reduction was studied by comparing the performance of HKUST-1, QH-200, QH-240, QH-260, and QH-300 at room temperature (Table 1 and Figure 6). After QH-*x* catalysts were added, the peak at 400 nm sharply decreased and finally disappeared, while a new peak grows at 300 nm, demonstrating the conversion of 4-NP to 4-AP (Figure 6). Depending on the thermolysis temperature, the synergetic combination of the uniform and extended distribution of active Cu(II) sites throughout the framework, prevention of particle agglomeration, and existence of both types of micro- and mesoporous structures are responsible for the enhancement of the catalytic activity. The relative catalytic activity of the HKUST-1 and QH-*x* samples also indicates that CuO (sample QH-300) is more active from the catalytic point of view than are framework octahedral Cu^2+^ ions (HKUST-1), while the presence of Cu_2_O (sample QH-260) increases the activity with respect to pure CuO. A QH-300 sample prepared with a shorter thermal treatment of 30 min instead of 120 min was also tested (Table 1) showing that a fast transformation of HKUST-1 into CuO takes place at this temperature. From Table 1, it can be concluded that tetrahedral Cu^2+^ ions together with some minor Cu^+^ ions prevalent in QH-240 are the most active sites for 4-NP reduction.

**Table 1:** Effect of Deligandation Temperature on the NP Reduction Process Catalyzed by Various Quasi-MOFs^a^

quasi-HKUST	reduction time (min)	reduction (%)	induction period (s)
HKUST	120	36	100
QH-200	100	51	60
QH-240	3	99.8	15
QH-260	40	95	40
QH-300	45	62	120
QH-300-30 min^b^	45	65	125

a Experimental conditions: *m*(catalyst) = 1.0 mg, [4-NP] = 0.05 mM, [NaBH_4_] = 10 mM, and *T* = 25 °C.

b QH-300-30 min was prepared as sample QH-300, but with 30 min of thermal treatment instead of 120 min.

As it can be seen in Table 1, the catalytic performance is enhanced by increasing the deligandation temperature up to 240 °C and decreasing afterward. QH-200 and QH-260 catalysts were less active than the QH-240 catalyst because the concentration of Lewis acid sites in QH-240 was found to be higher than that of the other catalysts, while at the same time QH-240 exhibits a distribution of both micro- and mesopore structures (Figure 3).

The deligandation of HKUST up to 200 °C mostly leads to the removal of solvent molecules without any salient change in the framework porous structure, thus resulting in a moderate increase in catalytic activity. The concentration of open metal sites increases in parallel with the mesopore volume of the framework as deligandation continues up to 240 °C, which causes a notable enhancement in the catalytic 4-NP reduction, requiring a much shorter time to achieve almost complete conversion. Upon further temperature increase, most of the framework collapses, resulting in a dramatic decline in surface area and, therefore, in catalytic activity.

Also, the induction period (*t*_0_), in which no catalytic reduction happens, decreases from 100 to 15 s in the presence of HKUST and QH-240 catalysts, respectively, while increasing afterward. Here, the induction time is attributed to the generation of the active reducing agent on the catalyst sites, and the 4-NP reduction reaction starts after this time.^35^ These observations can confirm that the restructuring of the catalyst sites proceeds more easily in the presence of QH-240. Therefore, the QH-240 sample was chosen as the best catalyst for additional studies on the catalytic 4-NP reduction.

It is known that NaBH_4_ can undergo hydrolysis in the presence of acid catalysts, releasing H_2_. This H_2_ evolution was also observed here in the absence of 4-NP. Interestingly, when using 4-NP the amount of H_2_ release is significantly diminished to about one-half in accordance with the occurrence of 4-NP reduction simultaneously with the H_2_ generation. Table S2 summarizes the amount of H_2_ measured after 5 h of reaction time.

**Figure 7:**
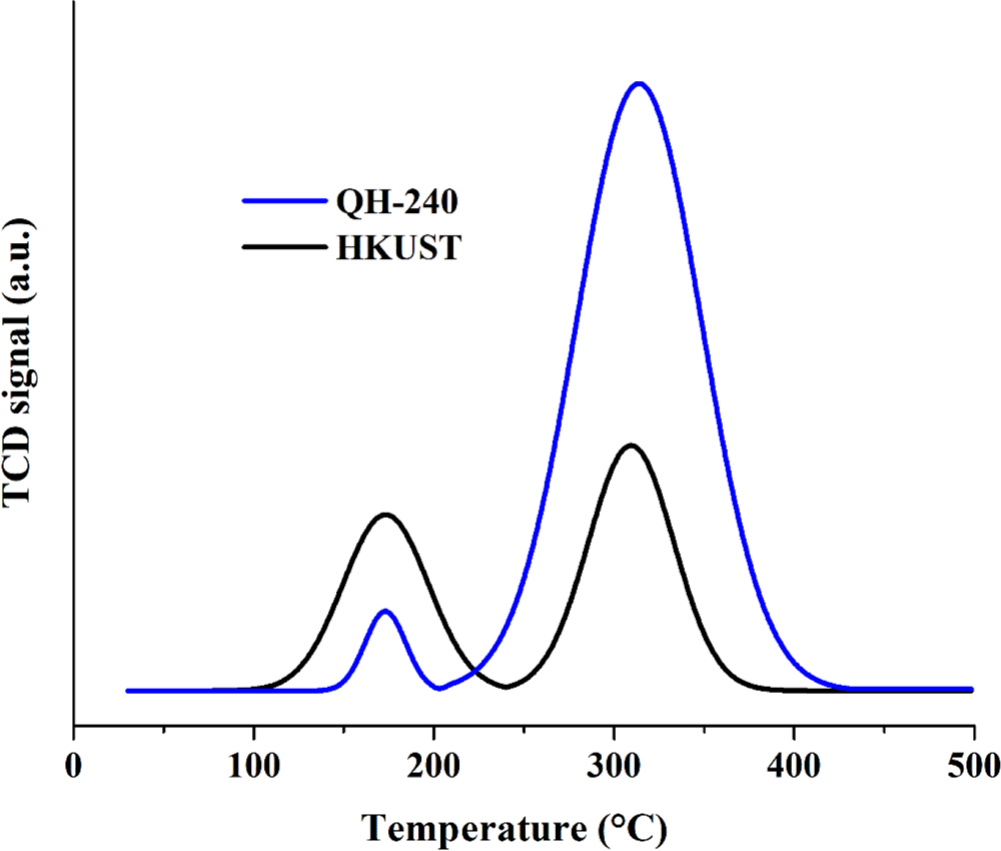
NH_3_-TPD analyses of (a) HKUST and (b) QH-240 catalyst.

The types and amount of acidic sites in the HKUST-1 parent and QH-240 catalysts were also assessed by temperature-programmed desorption of NH_3_ (Figure 7 and Table S3). Two NH_3_ desorption peaks were measured for HKUS at 170 and 303 °C, corresponding to sites of low and medium acid strength, respectively. For QH-240, these peaks appear at 172 and 313 °C. The much higher NH_3_ adsorption amount measured by TPD for QH-240 as compared to its parent HKUST-1 indicates that the thermal treatment generates an increase in the total population of Lewis acid sites of about 2.2 times, most of this increase being due to sites of medium strength. This increase in the population and strength of Lewis sites is one of the reasons explaining the higher catalytic activity of QH-240 in the 4-NP reduction. Worth noting is that the pH of the solution under the conditions of Table 1 is about 10. We performed an additional test of QH-240 catalytic activity at pH 8 under conditions otherwise identical to those of Table 1. A 4-NP conversion of 65% was achieved at pH 8, much lower than that at the NaBH_4_-free pH. This could be due to the reaction of NaBH_4_ with the H^+^ used to decrease the pH value, rendering H_2_ rather than promoting 4-NP reduction. When the pH is lower than 7, the 4-NP solubility is compromised, because 4-nitrophenolate is not formed.

**Figure 8:**
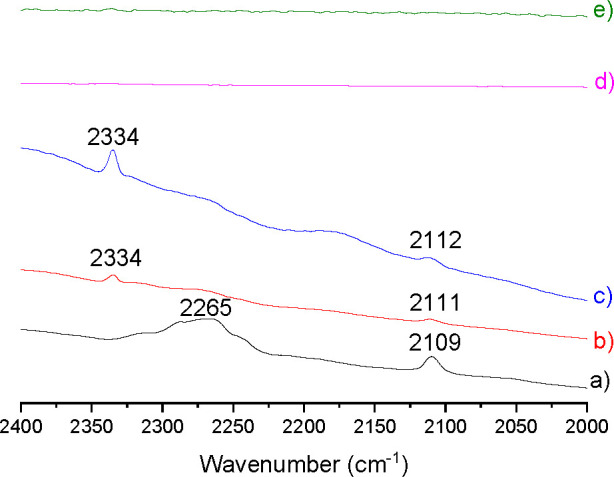
FT-IR spectra in the characteristic C≡N stretching region after adsorbing CD_3_CN on HKUST-1 (a), QH-240 (b), QH-260 (c), QH-300 (d), and QH-400 (e). The peak at 2334 cm^–1^ is associated with the C≡N interaction with Lewis acid sites of moderate strength, while the broad band at 2265 cm^–1^ corresponds to physisorbed acetonitrile.

Further characterization of the acid sites was performed by FT-IR spectroscopy monitoring the wavenumber of the characteristic C≡N vibration band of deuterated acetonitrile that is a common test of acidity. The results are presented in Figure 8. As it can be seen there, while HKUST-1 shows a broad and intense band in the 2265 cm^–1^ region characteristic of physisorbed CD_3_CN, QH-240 and QH-260 show a higher-frequency, sharp peak at 2334 cm^–1^, indicating the presence of Lewis acidity of medium strength. No CD_3_CN adsorption was observed for the QH-300 and QH-400 samples corresponding to Cu_2_O, which correlates well with their poor catalytic activity. The higher intensity of the 2334 cm^–1^ peak associated with Lewis acid sites interacting with acetonitrile for QH-260 as compared to QH-240 indicates that, besides acidity, other factors such as surface area and porosity are also playing a role. Note the large decrease in surface area measured from 160 m^2^ g^–1^ for QH-240 to 5.6 m^2^ g^–1^ for QH-260 (Table S1). In this way, QH-240 is the sample in which the best compromise among all of the factors has been achieved, resulting in the higher catalytic activity observed for QH-240 in comparison to QH-260 (see Table 1).

### Effect of QH-240 Catalyst Dosage

Because the amount of catalyst plays an important role on the reaction rate, the effect of various dosages of QH-240 catalyst on the room-temperature 4-NP reduction was also examined (Table 2 and Figure S17). No change in 4-NP concentration was observed at rt in the absence of catalyst.^34^ In addition, the reaction rate of 4-NP reduction increases continuously in the presence of increasing amounts of QH-240. The reduction performance reaches 98.3% within 6 min over 0.2 mg of QH-240. As the dosage of QH-240 catalyst increases up to 1.0 mg, the reduction efficiency reaches 99.8% at a shorter time of 3 min under the same reaction conditions. Afterward, the addition of increasing QH-240 amounts (>1.0 mg) does not result in a significant increase in the reduction rate under the present conditions. Thus, 0.2 mg is chosen as the suitable amount of QH-240 catalyst for subsequent catalytic experiments.

**Table 2:** Effect of the QH-240 Amount as a Catalyst on the Reduction Efficiency of 4-NP^a^

entry	catalyst dosage (mg)	4-NP conversion (%)	reduction time (min)^b^	4-AP yield (%)^c^
1	0	0	1 week	0
2	0.05	75	60	53.1
3	0.1	87	15	78.8
4	0.2	98.3	6	89.5
5	0.5	99.5	4	92.1
6	1.0	99.8	3	93.4

a Experimental conditions: [4-NP] = 0.05 mM, [NaBH_4_] = 10 mM, and *T* = 25 °C.

b Reduction time = time after subtraction of induction time for each catalytic run.

c The concentration changes of 4-AP obtained from the absorption changes in λ_max_ = 300 nm.

### Investigation of the Catalytic Reduction Kinetics

**Figure 9:**
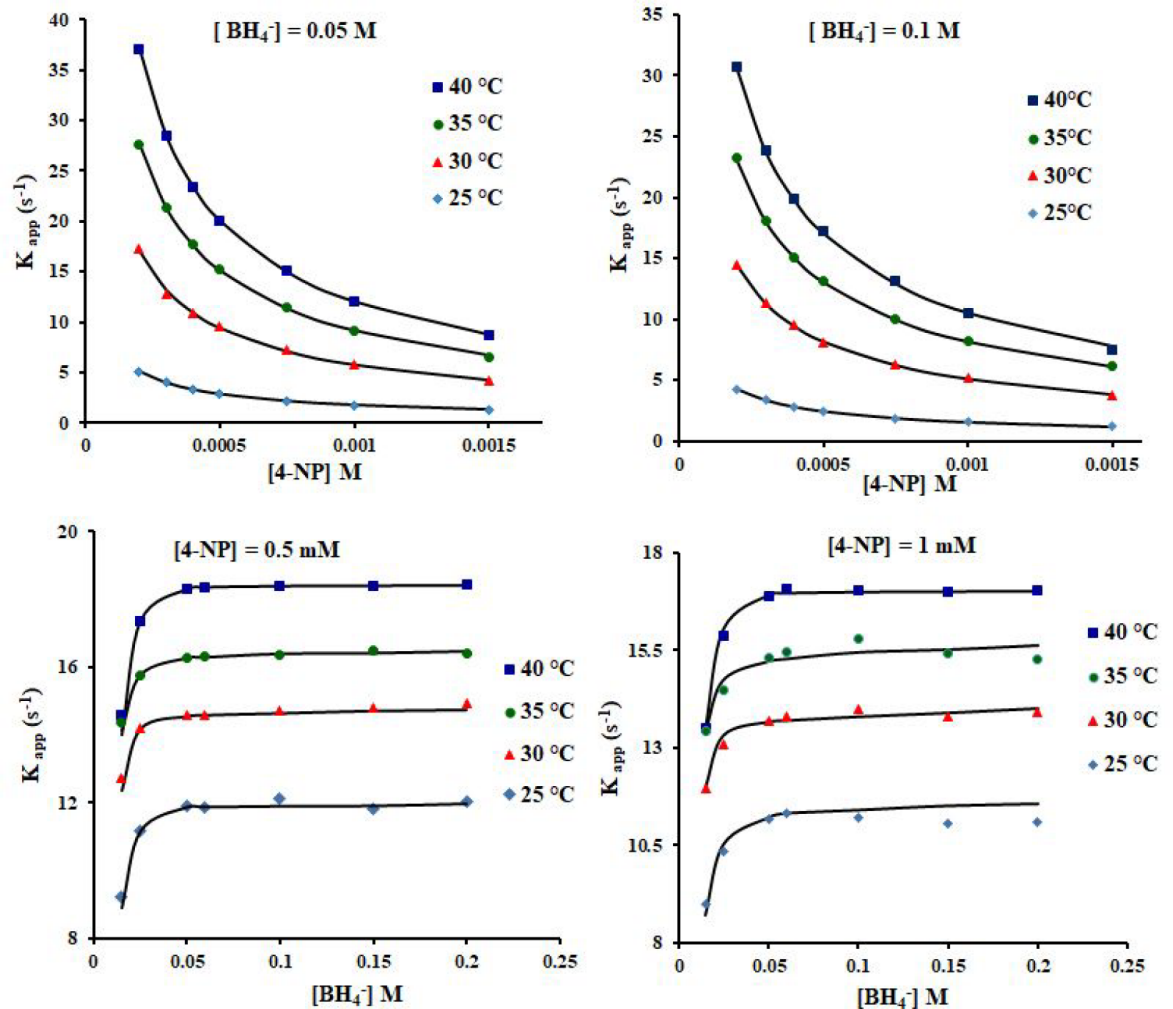
Dependence of the apparent rate constant (*k*_app_) on 4-NP and BH_4_^–^ concentrations at various temperatures. The black solid lines are the best fit of the experimental data to the LH model with the calculated surface area of QH-240 catalyst in solution of about 1.28 m^2^ L ^–1^.

Regarding the reaction kinetics, the key parameters such as the half-life time and the catalytic reaction order were measured both for HKUST and for QH-240 catalysts (Table 3). As expected, when the reaction is carried out in an excess of NaBH_4_ as reducing agent, the catalytic reduction of 4-NP was found to obey pseudo first-order kinetics using HKUST-1 or QH-240 as catalysts. The plots of ln(*C*/*C*_0_) versus time for the reduction of 4-NP using QH-240 and HKUST catalysts are provided in Figure S18. 4-NP concentrations higher than 0.05 mM were also tested (Figure 9). The pseudo first-order rate constant values (*k*_app_) at room temperature for HKUST and QH-240 catalysts were 3.0 × 10^–4^ and 1.02 × 10^–2^ s^–1^, respectively. QH-240 catalyst shows a higher activity factor of 51 s^–1^ g^–1^ at room temperature. This activity factor is 34 times higher than that of the HKUST catalyst (Table 3). Additionally, the half-life time of the catalytic reduction decreases from 38.5 to 1.13 min using the HKUST and QH-240 catalysts, respectively, which is 34 times faster when using QH-240 catalyst.

**Table 3:** Comparison of the Apparent Reaction Rate (*k*_app_) and Activity Factor (*K*) of the HKUST and QH-240 Catalysts for the 4-NP Reduction at Room Temperature

catalyst	*k*_app_ (s^–1^)	*t*_1/2_ (s)	*K* (s^–1^ g^–1^)
HKUST	3.0 × 10^–4^	2310	1.5
QH-240	1.02 × 10^–2^	68	51

The activity difference between the HKUST and QH-240 can be ascribed to the larger quantity of active sites as well as the higher mesopore volume in QH-240, resulting in a more favorable diffusion of reagents to a larger number of catalytic sites. On the other hand, the benzene ring of the ligand in the MOF mesopores can facilitate 4-nitrophenol adsorption via π–π stacking interaction and hence increase the catalytic efficiency (Figure S13). Therefore, the synergistic effect of the existence of both meso- and micropores (type I and IV curves) and the greater number of Lewis sites in the catalyst are probably responsible for the better adsorption of 4-NP and BH_4_^–^ ions, reducing the activation kinetic barrier that subsequently results in its superior catalytic performance in comparison with the parent HKUST-1.

### Reaction Mechanism, Rate Constants, and Adsorption Constants of 4-NP and BH_4_^–^ over QH-240

As was reported in the literature,^34,35,41^ a possible mechanism for the reduction of 4-NP by NaBH_4_ as reducing agent over a metal catalyst obeys the Langmuir–Hinshelwood (LH) model. In this model, the reaction proceeds having both BH_4_^–^ and nitrophenolate ions adsorbed on the surface of the catalyst, on which the transfer of a hydrogen species would occur. To achieve a complete collection of kinetic data, the apparent rate constant (*k*_app_) was calculated in a fixed concentration of 4-NP at a given temperature, while varying the NaBH_4_ concentration. In a second series of kinetic measurements, the concentration of NaBH_4_ was kept constant, but the 4-NP concentration was varied. Because the temperature is a main parameter in 4-NP reduction, the influence of the reaction temperature was also investigated (Table 4 and Figure 9).

**Table 4:** Rate Constants for 4-NP Reduction and Adsorption Constants of 4-NP and BH_4_^–^ at Different Temperatures on QH-240 Catalyst Determined from the Fitting of the Experimental Data to the LH Model According to Equation 7a^a^

temp (K)	*k* [mol/m^2^·s] × 10^2^	*K*_NP_ [L/mol]	*K*_BH_4__ [L/mol]
298	1.0 ± 0.1	2499 ± 130	89 ± 11
303	3.0 ± 0.1	3067 ± 175	92 ± 14
308	4.6 ± 0.1	3574 ± 250	93 ± 13
313	5.8 ± 0.1	4416 ± 265	95 ± 15

a *n* = 0.5, *m* = 1 Freundlich exponents in eq 7a.

The apparent rate constant is proportional to the surface area of the catalyst (*S*). The kinetic constant can be calculated as
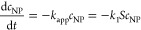
5

For quantitative data analysis, the catalytic reduction can be modeled by the Langmuir–Freundlich isotherm:
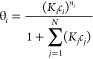
6where θ_*i*_ is the surface coverage of the compound *i*, *K*_*i*_ is the adsorption constant of the respective component, *c*_*i*_ is the concentration in solution, and “*n*” is related to the heterogeneity of the sorbent. Further rearrangement of eq 5 creates eq 7, which can be applied to model the catalytic activity.
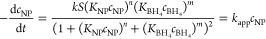
7

Thus, *k*_app_ is calculated by
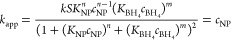
7aHere, *k* is the molar rate constant per square meter of the catalyst, and *K*_NP_ and *K*_BH_4__ are the adsorption coefficients of 4-NP and BH_4^–^_, respectively.

**Scheme 1:**
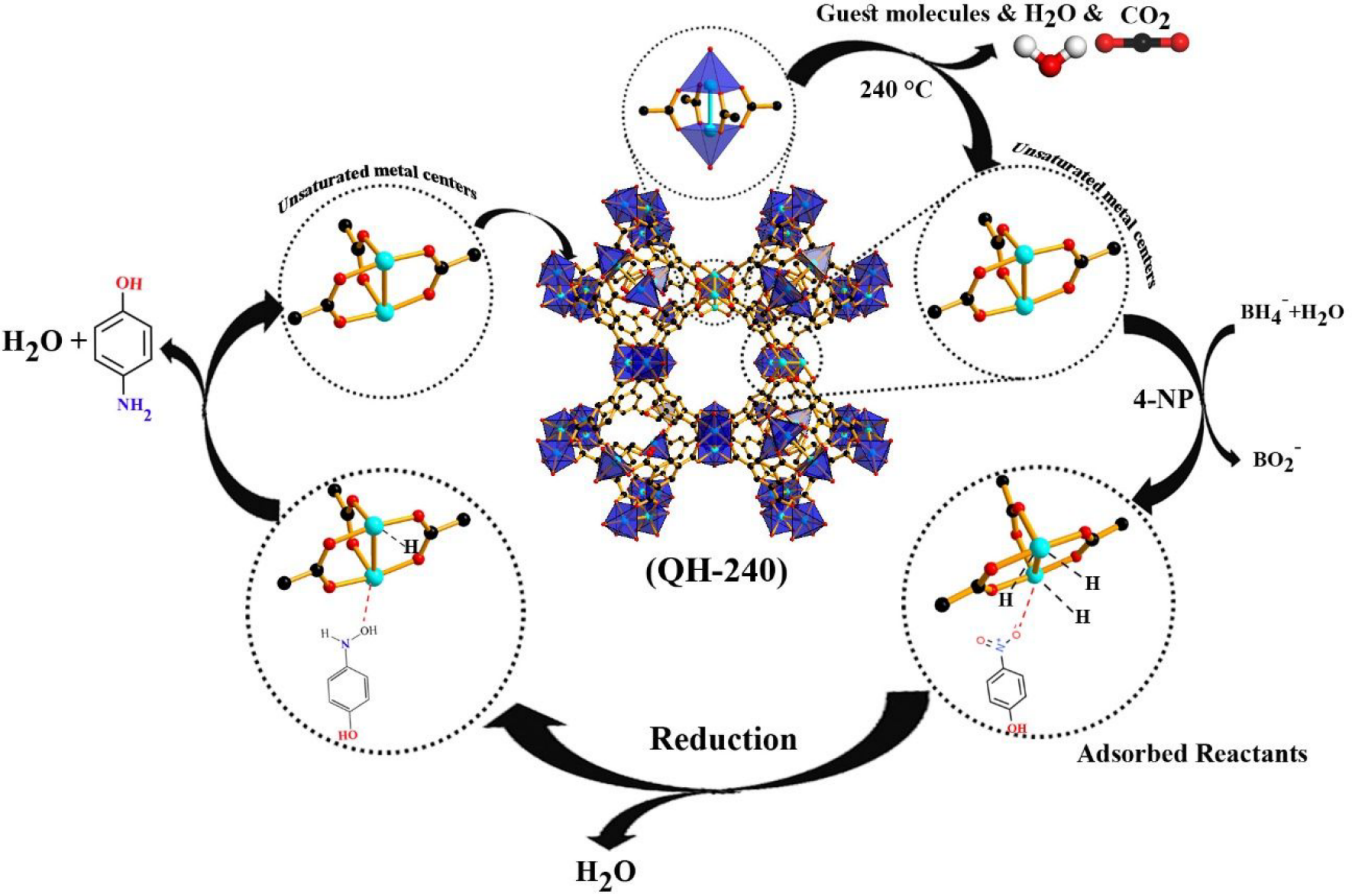
Mechanistic Proposal Based on the LH Model for the Reduction of 4-NP by NaBH_4_^–^ in the Presence of QH-240 Catalyst

According to eq 7, an almost complete coverage of the catalyst surface by 4-NP occurs in a high concentration of 4-NP molecules, causing a dramatic decrease in the reaction rate due to the absence of BH_4_^–^ ions on the active metal sites (Figure 9). In addition, the dependence of *k*_app_ on BH_4_^–^ concentration is nonlinear (Figure 9), and saturation of *k*_app_ at high BH_4_^–^ concentrations indicates that there must be a competition of both reactants for the active Cu sites on the catalytic surface. These observations agree with the operation of the LH model for the catalytic 4-NP reduction with QH-240 as catalyst. The rate constant decreases by increasing the concentration of 4-NP and increases by increasing the concentration of NaBH_4_, according to their relative adsorption constants. It is obvious that there is a competition between both reactants for active copper sites on the QH-240 surface. The diffusion of the reactants to the unsaturated Cu sites of QH-240 catalyst and the adsorption/desorption equilibrium are surmised to be fast. In the rate-determining step, the surface hydrogen and 4-NP react with each other, and the resulting 4-AP desorbs from the surface of the QH-240 catalyst. Separation of the produced 4-AP releases a free catalytic site, and a new turnover can occur again. A high concentration of 4-NP may cause the full coverage of the catalytic sites, which decreases the reaction rate. Increasing concentrations of NaBH_4_ provide an increase in the rate constant until a maximum rate constant is achieved. Beyond this point, an additional NaBH_4_ concentration increase decreases the rate constant due to the saturation on the catalyst sites. According to the results, both BH_4^–^_ ions and 4-NP simultaneously are adsorbed on the surface of the QH-240 catalyst, and then the produced 4-AP is desorbed out to regenerate the active site (Scheme 1). The adsorption constant of 4-NP is higher than that for BH_4_. Also, *K*_4-NP_ increases upon increasing temperature, while the *K*_BH_4__ is comparatively much smaller than the *K*_4-NP_ at each temperature in the range studied (Table 4). Confirmation of the strong 4-NP adsorption on QH-240 was also supported by observation in the IR of blue shifts in the frequency of the characteristic ligand vibrations from 1611 and 1542 to 1614 and 1551 cm^–1^, respectively, upon adsorption of 4-NP (Figure S13). These adsorption data are compatible with the initial quick reduction of 4-NP to the 4-hydroxylaminophenol intermediate that subsequently is slowly reduced to the final 4-AP product. That means the second step (*K*_BH_4_^–^_) is the rate-determining step.

Thermodynamic parameters for the heterogeneous catalytic reduction of 4-NP over QH-240 catalyst were determined by performing the reduction reaction at different temperatures from 25 to 40 °C, obtaining the true (*k*) rate constant at each temperature. The true activation energy (*E*_A,*k*_) was calculated to be 28.8 kJ/mol by the Arrhenius equation:
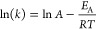
8

With these kinetic data, the activation enthalpy (Δ*H*^⧧^) and activation entropy (Δ*S*^⧧^) were also estimated by the Eyring equation:
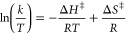
9

The Gibbs free energy of activation Δ*G*^⧧^ at 25 °C was also calculated. As presented in Table 5, the adsorption process for the 4-NP molecule is endothermic, while the activation entropy is a positive value, indicating the release of water molecules and/or other adsorbed species bonded to the catalyst surface, which results in an increase of entropy.

**Table 5:** Activation Parameters for the Adsorption of 4-NP and BH_4_ on the Surface of QH-240 Catalyst

parameters	*K*_4-NP_	*K*_BH_4_^–^_
Δ*H*^⧧^ (kJ/mol)	25.7 ± 0.2	1.7 ± 0.5
Δ*S*^⧧^ (J/mol·K)	106 ± 0.6	7.8 ± 0.4
Δ*G*_298_^⧧^ (kJ/mol)	–31.6 ± 0.4	3.2 ± 0.2

The negative Δ*G*_298_^⧧^ value of 4-NP adsorption indicates that the process occurs spontaneously in the presence of this reactant. Also, in the case of NaBH_4_, the Δ*G*_298_^⧧^ value is positive, but small, showing that the catalytic conversion is feasible over QH-240 catalyst at room temperature as observed experimentally. The activity factor of the QH-240 catalyst indicates that it is able to contend favorably with diverse reported copper base catalysts (Table 6). Also, the turnover frequency of the QH-240, which is defined as [(moles of 4-NP reduction)/(moles of active sites) × *t* (min)], is at about 0.73 min^–1^, considering tetrahedral Cu^2+^ ions as active sites in QH-240.

**Table 6:** Comparison of the Activity Factor of QH-240 with Other Reported Copper-Based Catalysts for the Conversion of 4-NP to 4-AP at Room Temperature

catalysts	*K*_app_ (s^–1^)	*K* (s^–1^ g^–1^)	*E*_a_ (kJ/mol)	catalyst dosage (mg)	NaBH_4_ (mol)/4-NP (mol)	ref
Cu_*x*_O@C-400	4.8 × 10^–3^	2.4		2	62.5	(42)
(Au_0.3_Pt_0.3_Pd_0.4_)/Cu(HBTC)-1	8 × 10^–3^	40		0.2	25	(43)
C@Cu	5.9 × 10^–2^	59		1	232	(44)
CuO	1.3 × 10^–3^	0.26		5		(45)
CuO/Fe_3_O_4_	3.6 × 10^–3^	0.72				
carbon-doped CuO/Fe_3_O_4_	6.5 × 10^–3^	1.3				
carbonized HKUST/melamine	1.8 × 10^–2^	1.8	73.6	0.1	200	(46)
Ag NPs@ZrGP^a^	1.7 × 10^–1^	17		10	100	(47)
QH-240	1.02 × 10^–2^	51	28.8	0.2	200	this study

a ZrGP: zirconium glyphosate.

### Reusability of QH-240 Catalyst

**Figure 10:**
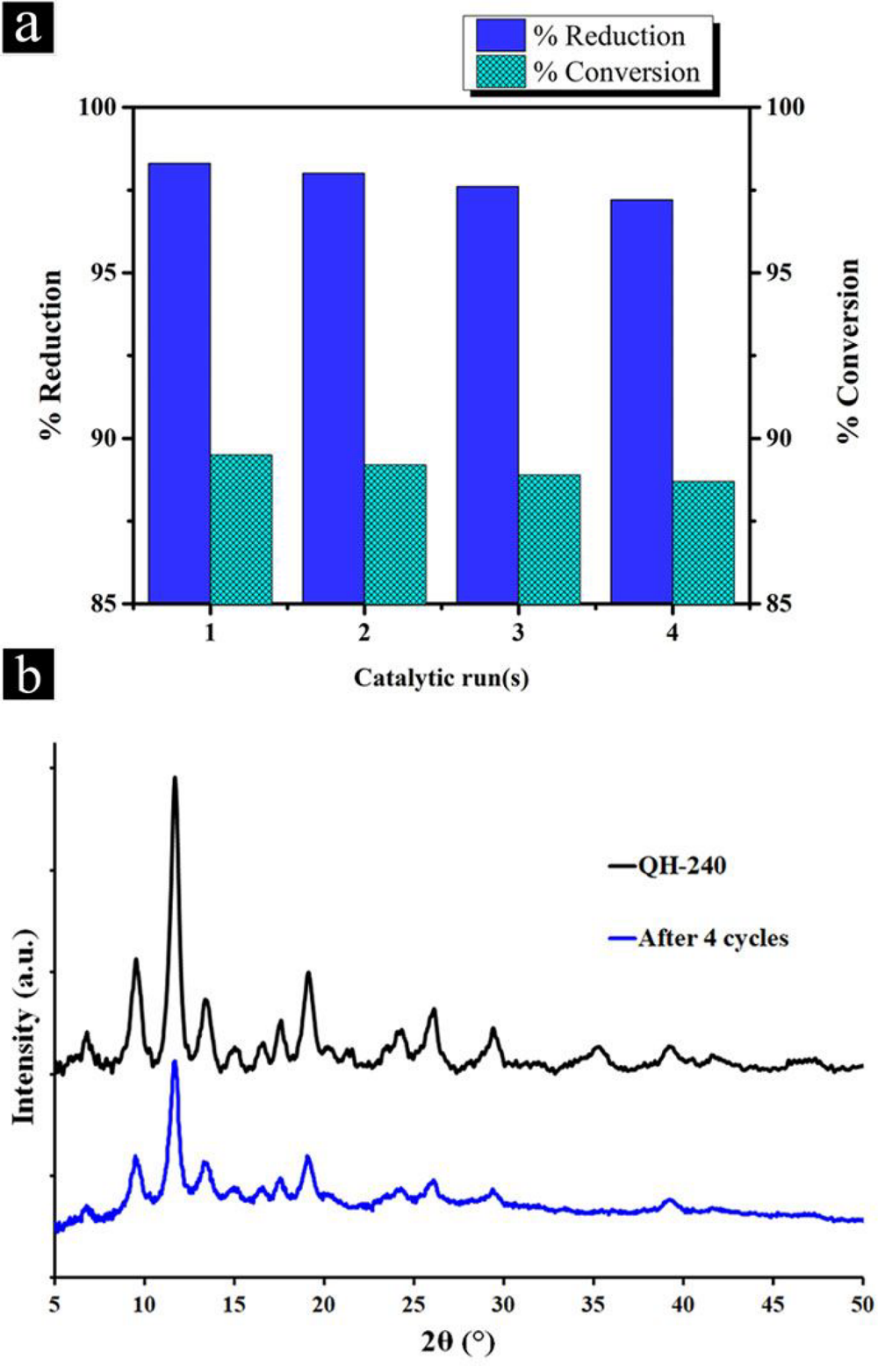
(a) 4-NP reduction and 4-AP conversion over QH-240 catalyst for four consecutive reuses. Experimental conditions: *m*_cat_ = 0.2 mg, [4-NP] = 0.05 mM, [NaBH_4_] = 10 mM at room temperature for a reaction time of 6 min. (b) XRD pattern of QH-240 catalyst after four catalytic cycles.

To examine catalyst reusability, the reduction of 4-NP over QH-240 was carried out for four consecutive runs, calculating the apparent rate constants for each run. The rate constants remain relatively unaltered, even for the fourth run. QH-240 catalyst exhibited a 4-AP yield of up to 98.3% and was recycled with no apparent loss of performance (Figure 10). Characterization by XRD of the QH-240 sample after the fourth use shows that the structure of the material remained unchanged (Figure 10b), indicating the stability of the catalyst. Moreover, ICP analysis indicates that the Cu leached from the solid in the aqueous supernatant after each cycle is not observed. These results prove that the QH-240 is stable and can be reused in the catalytic reduction of 4-NP.

### Comparison of the Catalytic Activity of QH-240

As commented in the Introduction, the purpose of the present study is to determine to which extent the catalytic activity of pristine MOFs, such as HKUST-1 in the present case, can be increased by thermal deligandation in quasi-MOFs. The previous reported studies provide relative data among HKUST-1 and the various quasi-MOFs derived therefrom. To put the activity achieved for QH-240 in a broader context, Table 6 compares the reported activity of other catalysts with that achieved herein for QH-240. The reaction under study is suitable for this comparison, because the first-order kinetics allows one to use the apparent first-order rate constant and its specific value activity factor as figures of merit. Although comparisons have always to be taken cautiously due to the possible influence of different factors and conditions, the data shown in Table 6 indicate that QH-240 is among the best material ever reported for this process, thus illustrating the potential of the thermal treatment for the optimization of MOF catalytic activity.

## Conclusions

Thermal treatment of HKUST-1 at temperatures between 200 and 300 °C may cause the partial decomposition of the BTC ligands resulting in a “quasi”-MOF material in which the crystal HKUST structure is still partially preserved on the way to CuO. Quasi-HKUST-1 structures act as non-noble hydrogenation catalysts for 4-nitrophenol reduction at room temperature. The higher activity of QH-240 derives from the synergistic effects of the increase in the density of unsaturated copper sites and the existence of both meso- and micropores in the catalyst. The strong adsorption coefficient of 4-NP on QH-240 suggests that phenyl rings of the partially damaged BTC ligands in the MOF mesopores still facilitate 4-NP uptake via π–π stacking, as supported by the blue shifts in the IR vibration frequencies of the benzenetricarboxylate ligands. Thus, the present results show that postsynthetic defect engineering via controlled thermolysis of MOFs is a simple and convenient way to further enhance the catalytic activity of MOFs that can be used for the removal of organic pollutants from contaminated waters to convert them into valuable side products.

## Supporting Information Available

FT-IR, Raman, EPR, and UV–vis spectra, SEM images, kinetics plots, and H_2_ evolution data (PDF)
