# Supplementary material for: Quasi-HKUST Prepared via Postsynthetic Defect Engineering for Highly Improved Catalytic Conversion of 4-Nitrophenol
Source: ACS Appl Mater Interfaces. 2021 Dec 31;14(1):978–89. doi: 10.1021/acsami.1c19862 (PMC8762642; doi:10.1021/acsami.1c19862)
Supplement: Supplementary file 1 — am1c19862_si_001.pdf [file am1c19862_si_001.pdf]

Supporting Information

Quasi-HKUST Prepared via Post-Synthetic Pore Engineering for Highly Improved Catalytic Conversion of 4-Nitrophenol

Minoo Bagheri,[a] Arianna Melillo,[b] Belen Ferrer,[b] Mohammad Yaser Masoomi*,[a] and Hermenegildo Garcia*,[b]

[a] Department of Chemistry, Faculty of Science, Arak University, Arak 38156-8-8349, Iran

Email: m-masoomi@araku.ac.ir

[b] Instituto Universitario de Tecnología Química Consejo Superior de Investigaciones Científica.-Universitat Politecnica de Valencia and Departamento de Química, Universitat Politecnica de Valencia, Av. De los Naranjos s/n, 46022 Valencia, Spain. Email: hgarcia@upv.es

Materials and Characterization Techniques.

All reagents used in the HKUST-1 synthesis and analysis of the reaction mixtures were commercially available (Aldrich and Merck Company) and used as received. IR spectra were recorded using Thermo Nicolet IR 100 FT-IR spectrophotometer. The thermal behavior was determined with a PL-STA 1500 instrument heating at a rate of 10 ºC min^{-1} under static N_2 atmosphere. Powder X-ray diffraction (PXRD) patterns were acquired with a Philips X'pert diffractometer with monochromatic Cu-K_{α} radiation. Surface area and porosity were performed using the TriStar II 3020 gas adsorption apparatus from Micrometrics Instrument Corporation and BELSORP-mini II from LMS Instruments Co., Ltd.. N_2 adsorptions were carried out at 77 K. Samples were heated before adsorption at 120 °C for 14 h under vacuum. Pore-size distributions were determined from the N_2 adsorption branch according to the BJH algorithm. X-ray photoelectron spectroscopy (XPS) measurements were performed on a BesTec (Germany) X-ray photoelectron spectrometer operating with an Mg K_{α} source. The particle morphology was analyzed with field emission scanning electron microscopy (FE-SEM) using a TESCAN MIRAIII (Czech) instrument. Possible Cu leaching to water from QH-240 sample during the reaction was analyzed by inductively coupled plasma (ICP) at the end of the $NaBH_4$ reduction of 4-NP. ICP-optical emission spectrometry (ICP-OES) analysis were done with a Varian Vista-PRO (Springvale, Australia) with a radial torch coupled to a concentric nebulizer, a Scott spray chamber and equipped with a charge-coupled detector (CCD).

TPD-TPR 2900 system equipped with a thermal conductivity detector (TCD) were used to perform thermoprogrammed adsorptions and reductions. First, a given catalyst amount was pretreated at 150 °C in a He stream under 20 mL min^{-1} for 1h. Afterwards, the system is cooled to 40 °C, and exposed to ammonia (4 vol.% in He, 20 mL min^{-1}) for 1 h. Physisorbed ammonia

was removed by flushing the solid with 20 mL min $^{-1}$ of He for 30 min. Eventually, the desorption was performed by raising the temperature from 40 to 750 °C at a heating rate of 10 °C min $^{-1}$ in He stream (20 mL min $^{-1}$).

Synthesis of HKUST-1 [$Cu_3(BTC)_2(H_2O)_3]_n \cdot nH_2O$MeOH

In a typical experiment HKUST was obtained by mixing BTC 2.1 g, 10 mmol) and $Cu(OAc)_2.H_2O$ (3.21 g, 16 mmol) in 400 ml H_2O:EtOH (50:50) in a round bottom flask at 110 °C for 4 h (See Supporting Information for more information). The resulting powder was isolated by centrifugation, washed four times with ethanol and four times with water and dried in air Yield: 3.185 g (91 % based on BTC). IR data (KBr pellet, ν/cm^{-1}): 485(w), 733(m), 756(m), 1108(w), 1374(s), 1463(s), 1557(s), 1615(s), 1656(s), 1702(m) and 3417(w-br) (Figure S1).

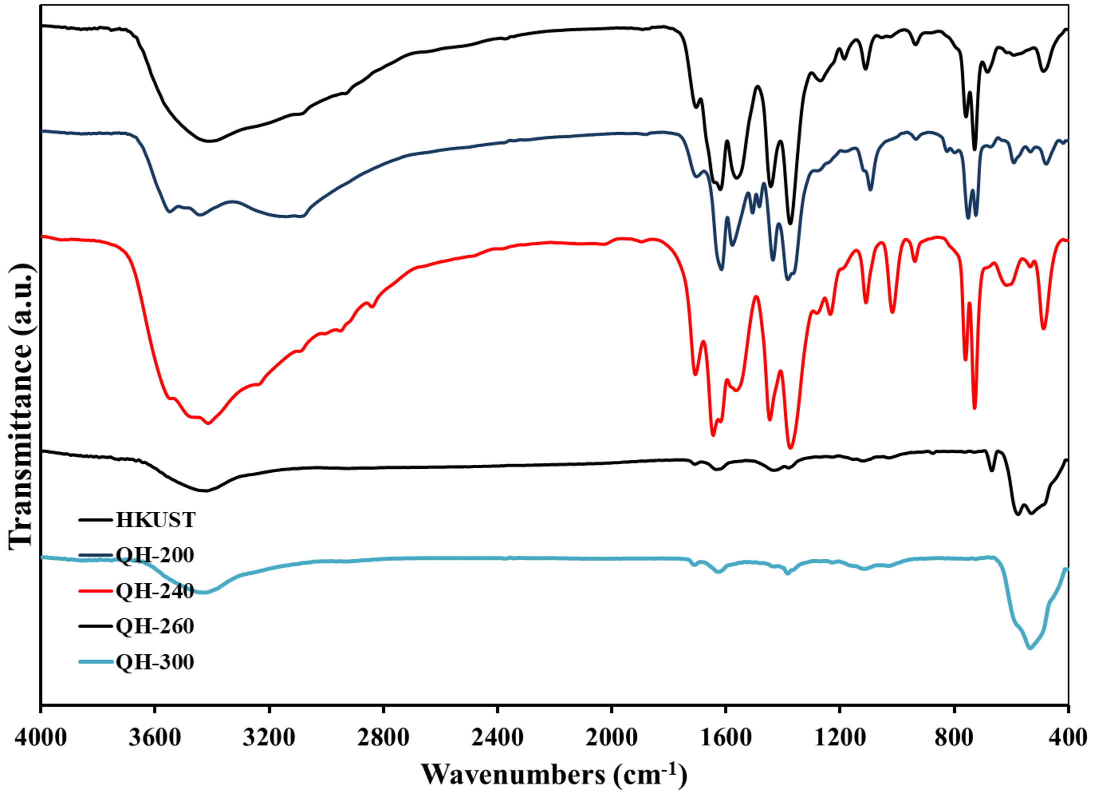

Figure S1. IR spectra of HKUST-1 and QH-x samples.

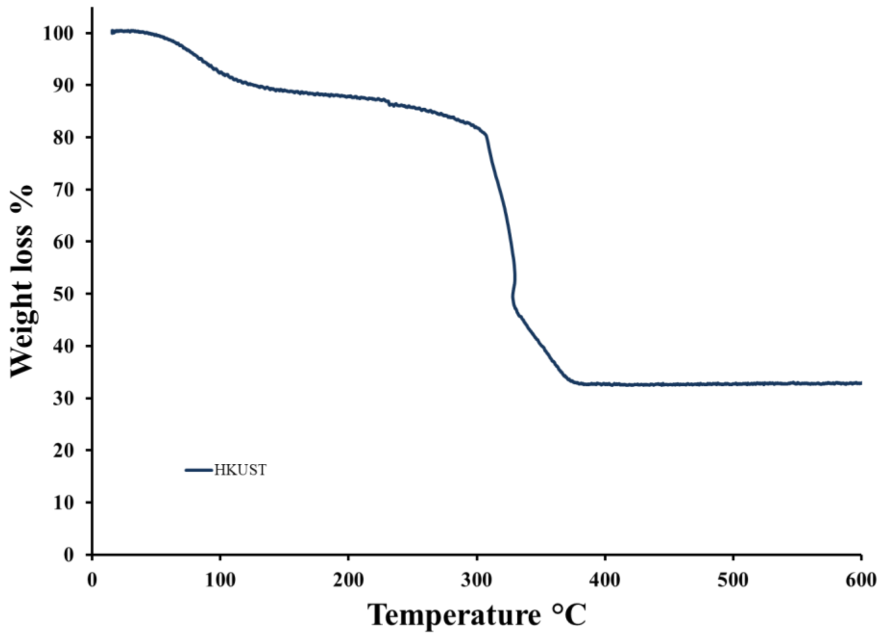

Figure S2. Thermogravimetric profiles of HKUST-1.

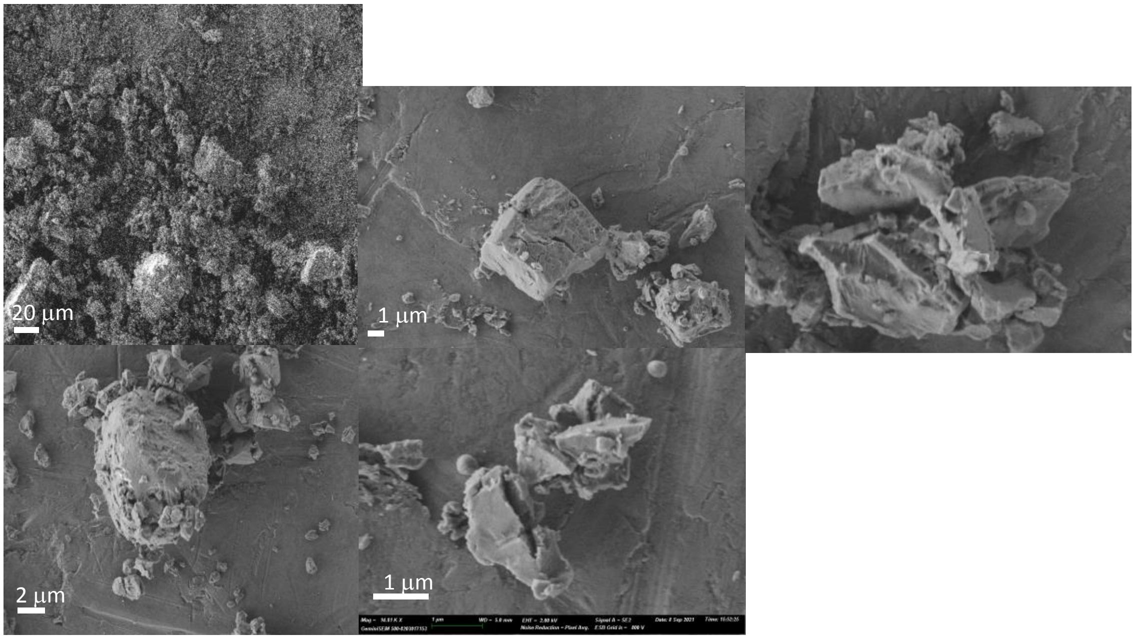

Figure S3. FE-SEM images of HKUST-1.

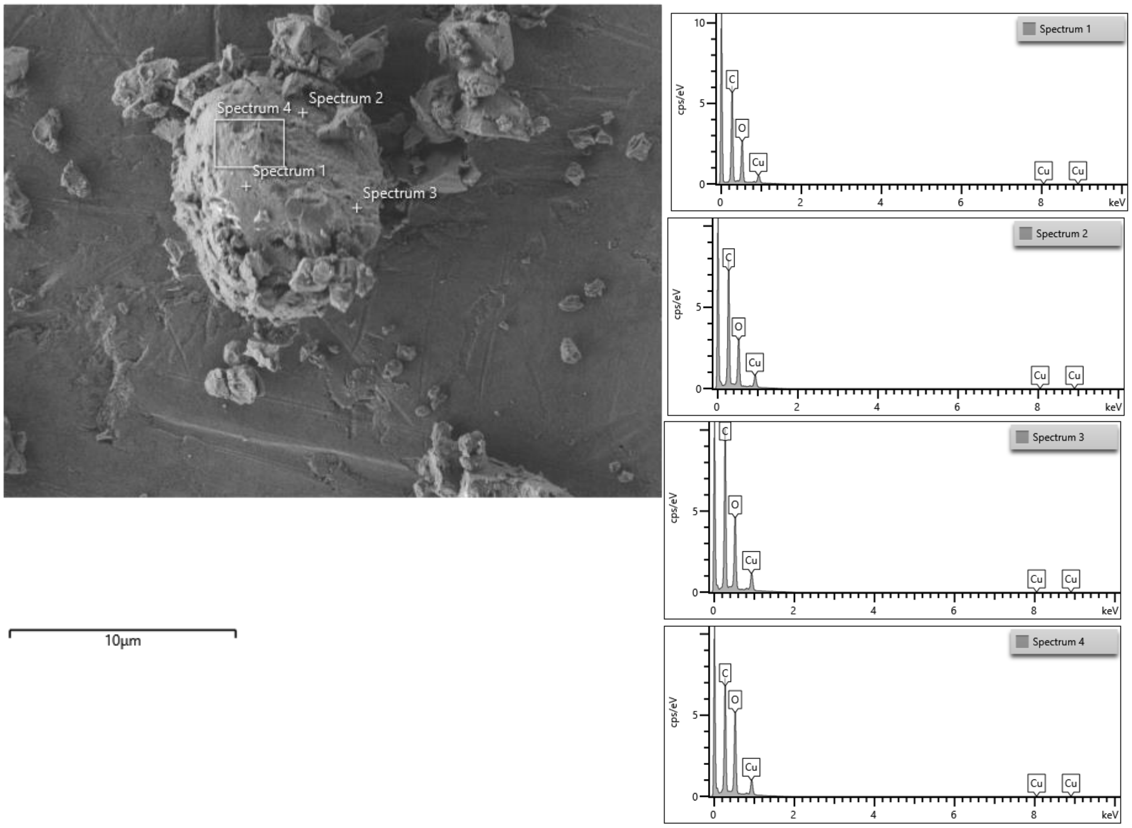

Figure S4. SEM image and elemental EDX mapping of HKUST-1.

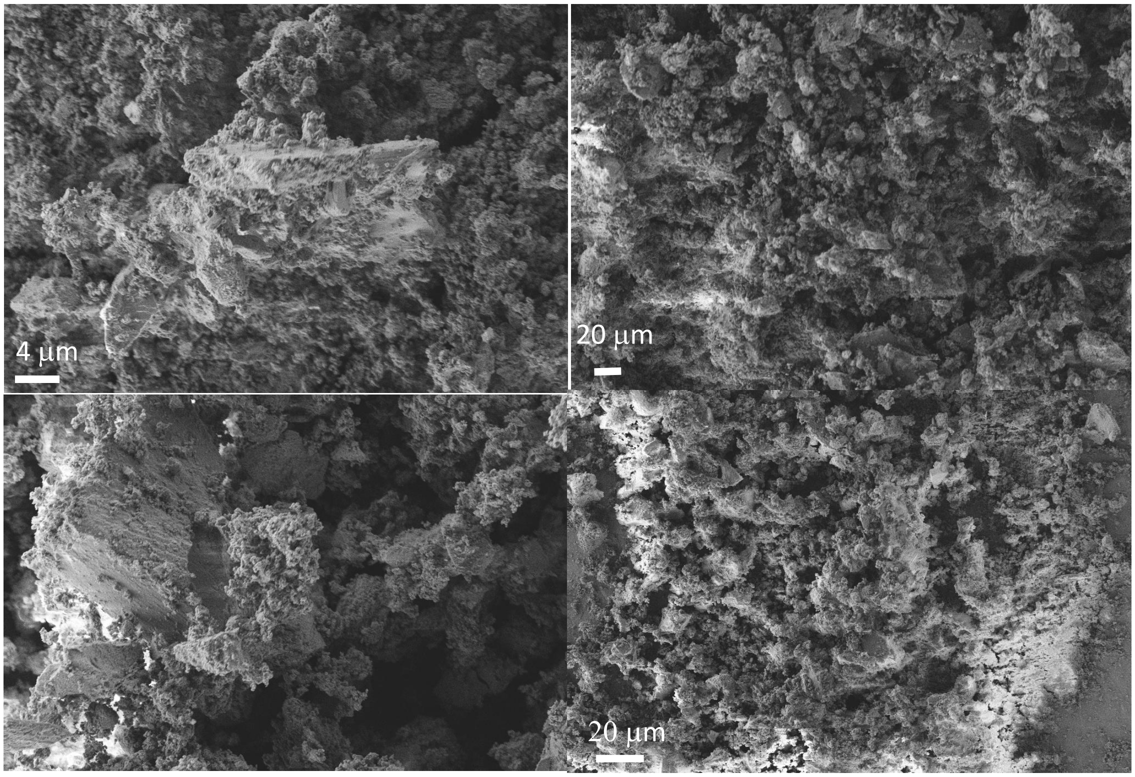

Figure S5. FESEM images of QH-240.

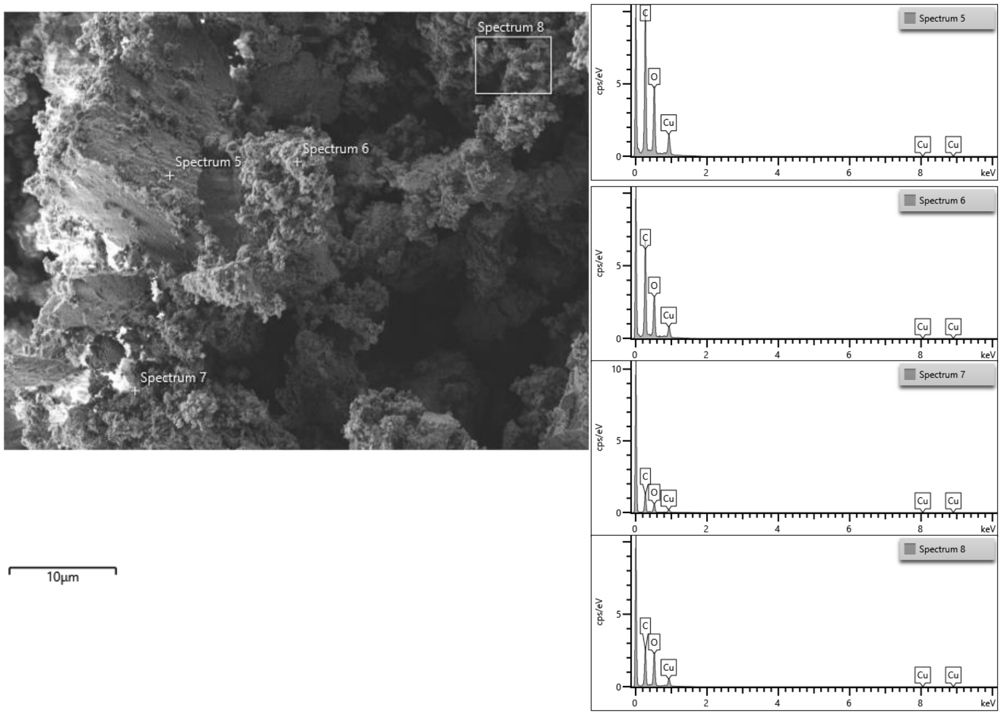

Figure S6. SEM image and elemental EDX mapping of QH-240.

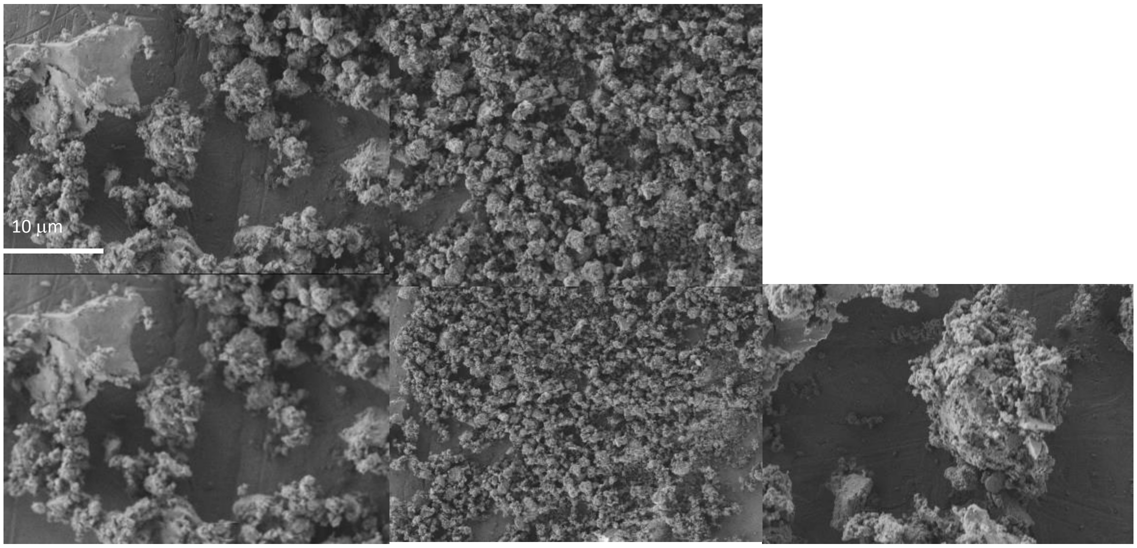

Figure S7. FESEM images of QH-260.

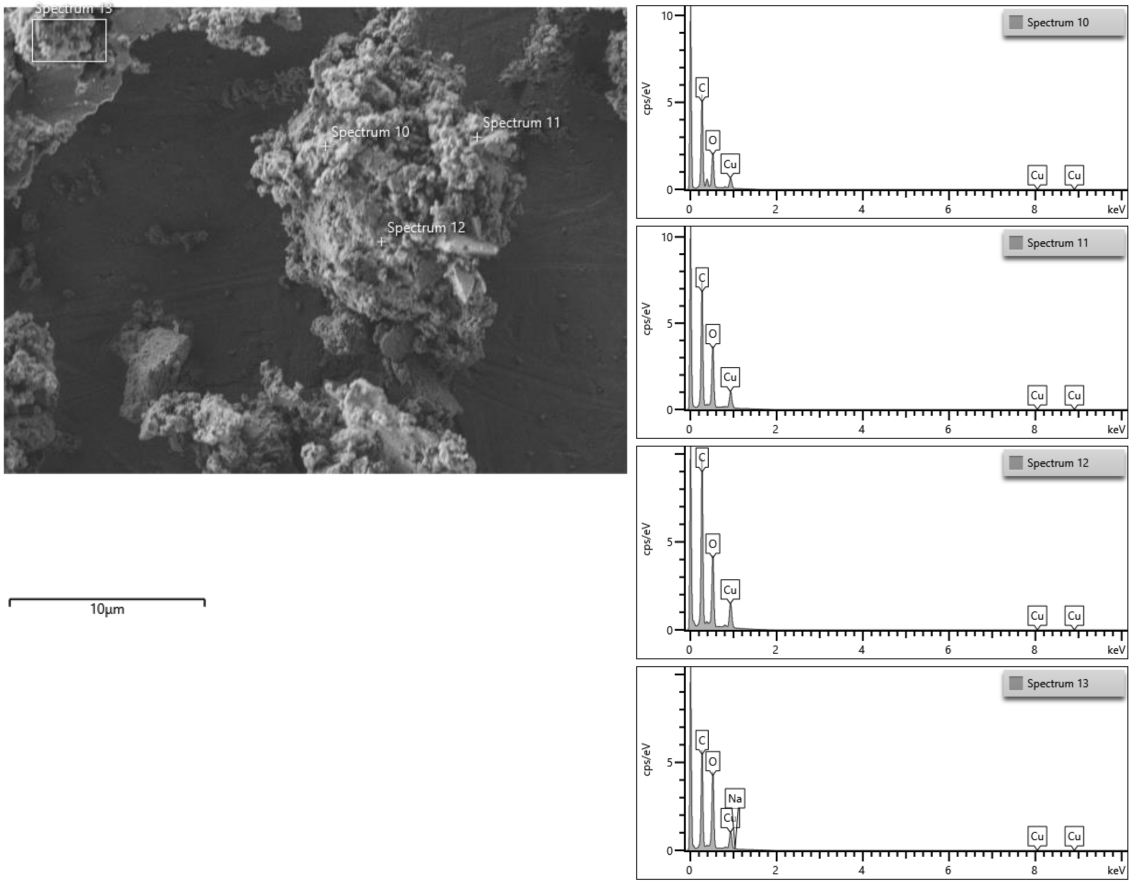

Figure S8. SEM image and elemental EDX mapping of QH-260.

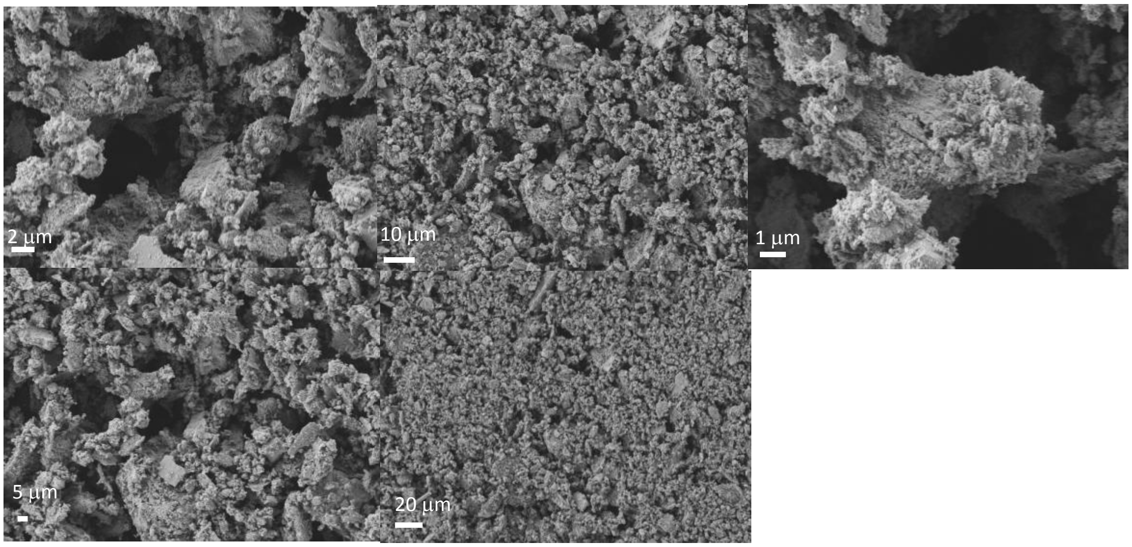

Figure S9. FESEM images of QH-300.

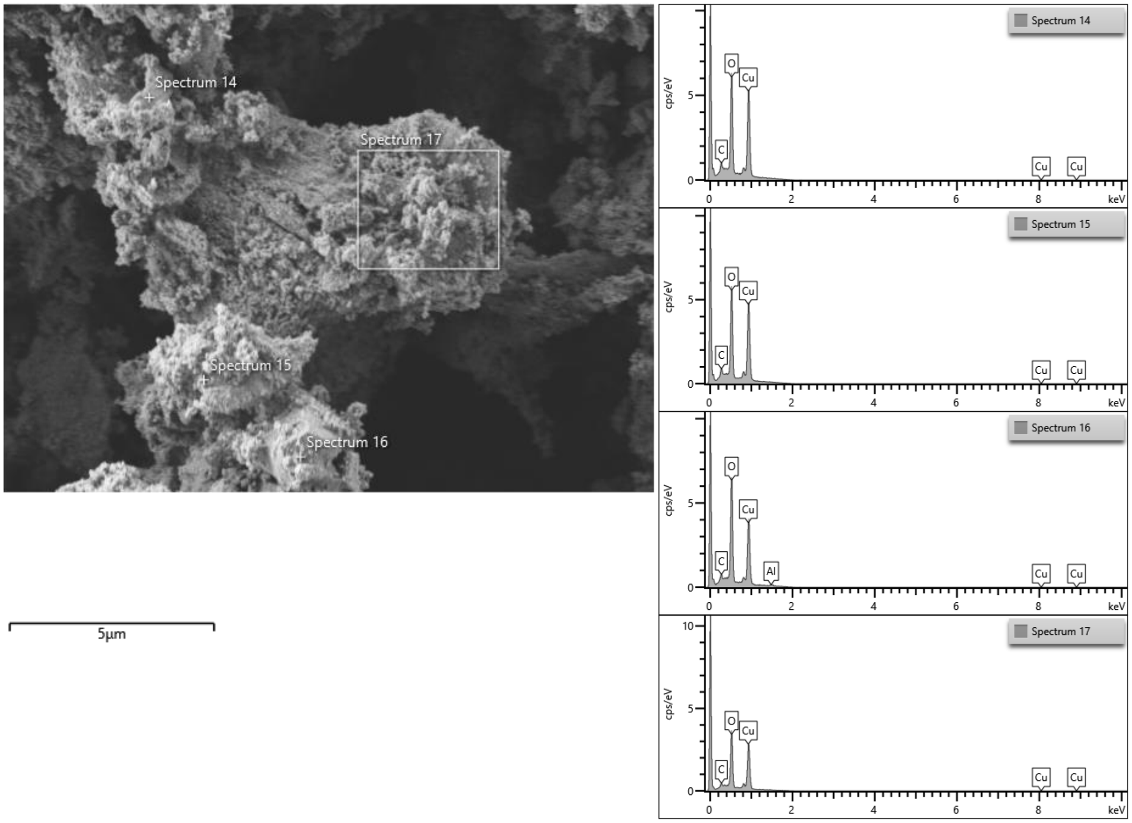

Figure S10. SEM image and elemental EDX mapping of QH-300.

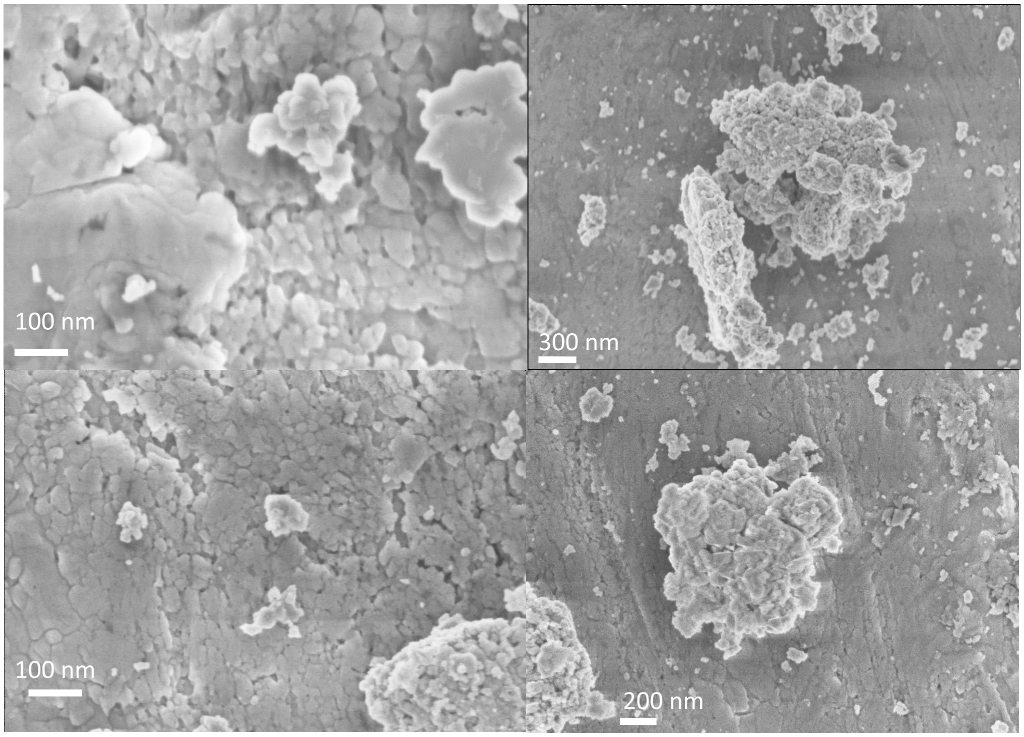

Figure S11. FESEM images of QH-400.

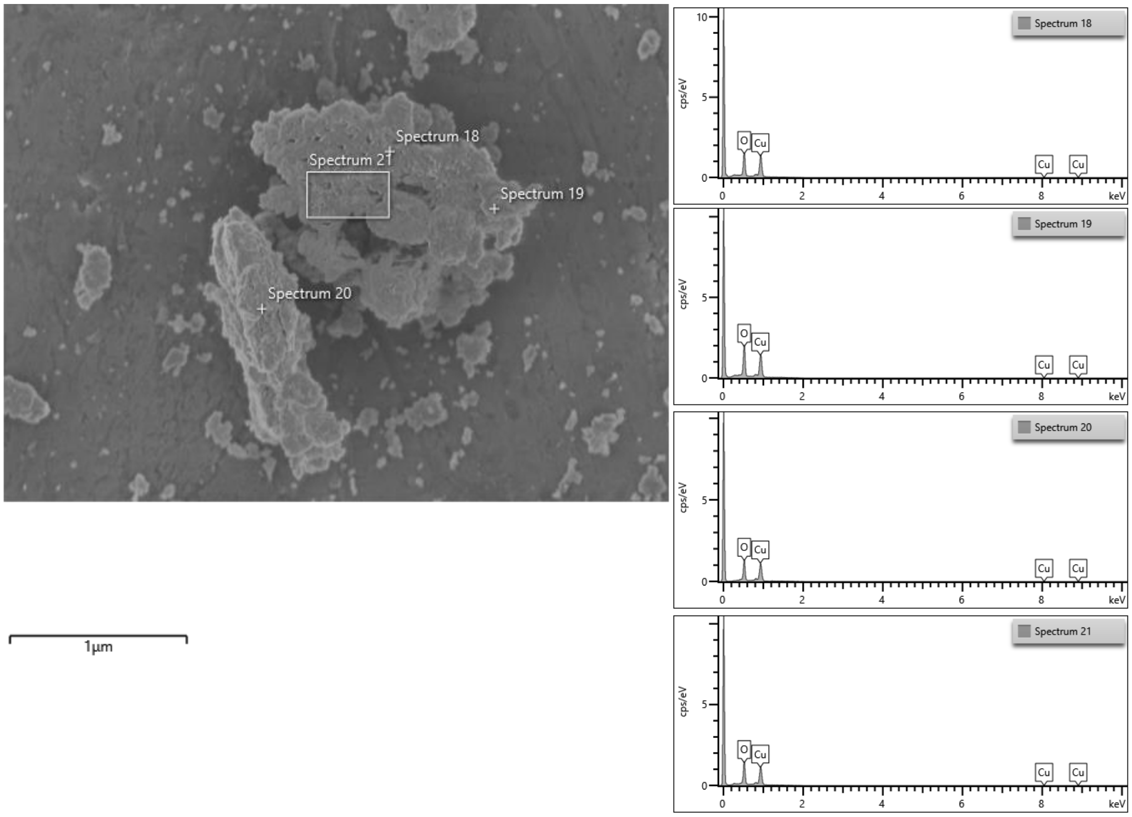

Figure S12. SEM image and elemental EDX mapping of QH-400.

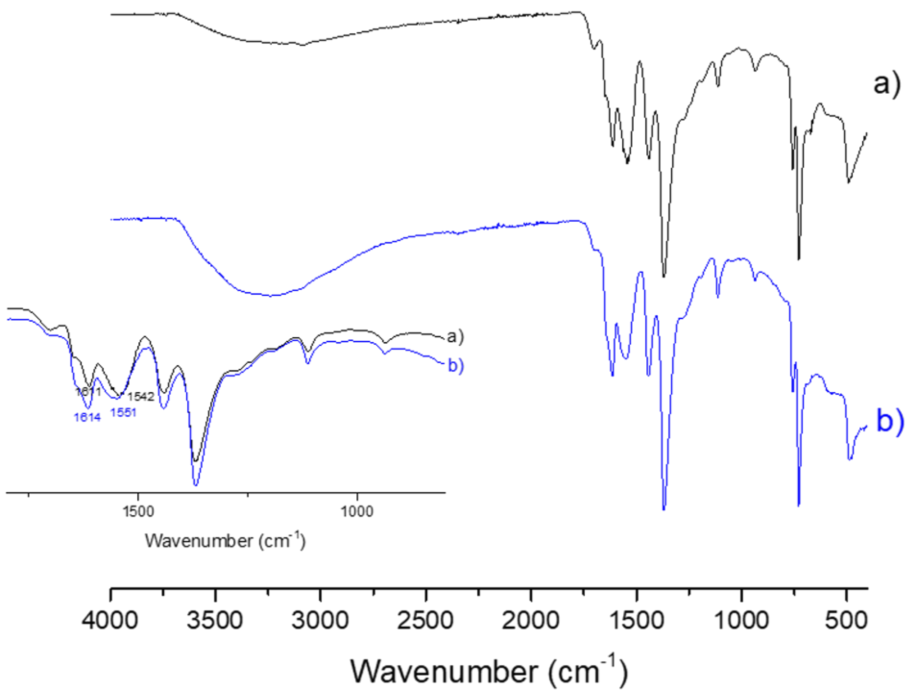

Figure S13. ATR analysis of a) QH240 and b) QH 240 after adsorption of 4-NP; the inset show a magnification of the 1800 and 800 cm^{-1} region.

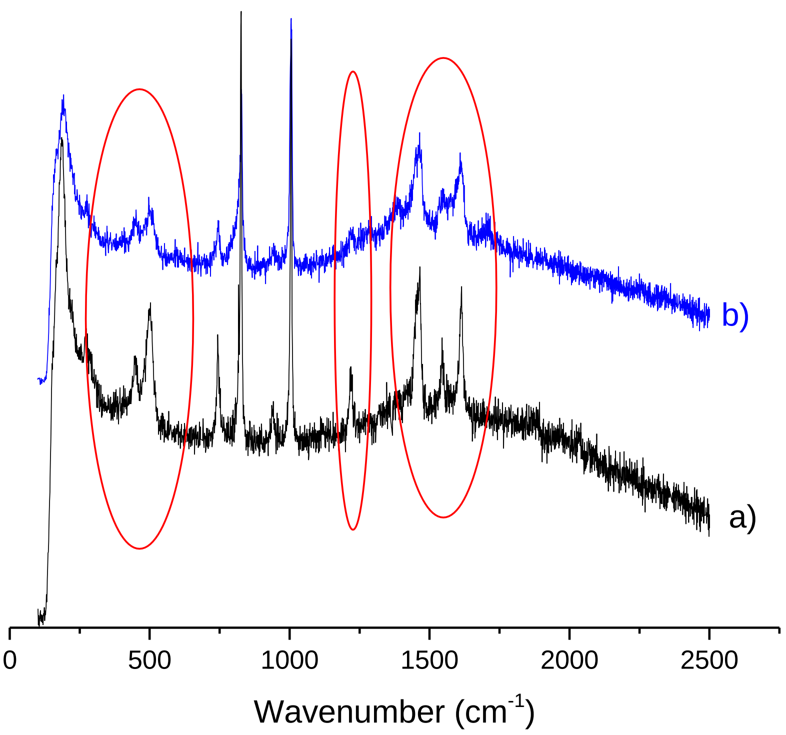

Figure S14. Raman data collected from a) HKUST-1 and b) QH-240.

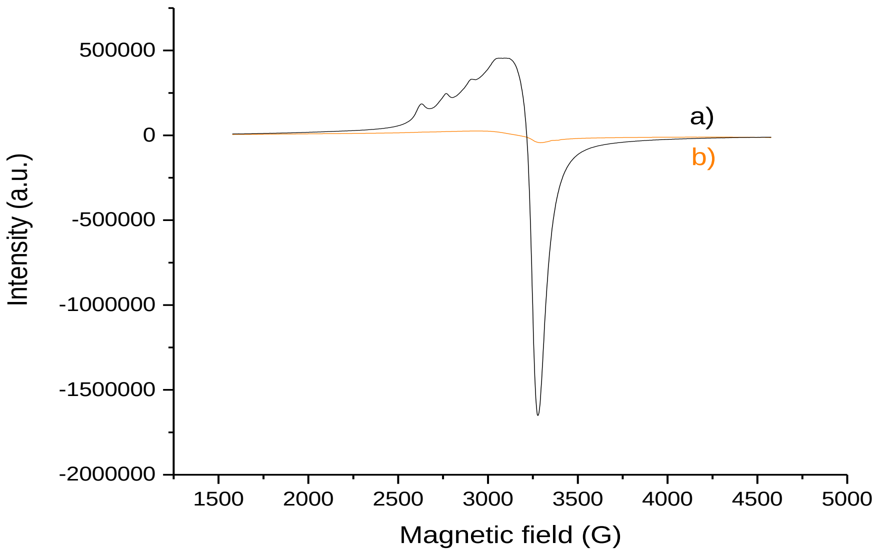

Figure S15. EPR data of a) HKUST-1 and b) QH 240

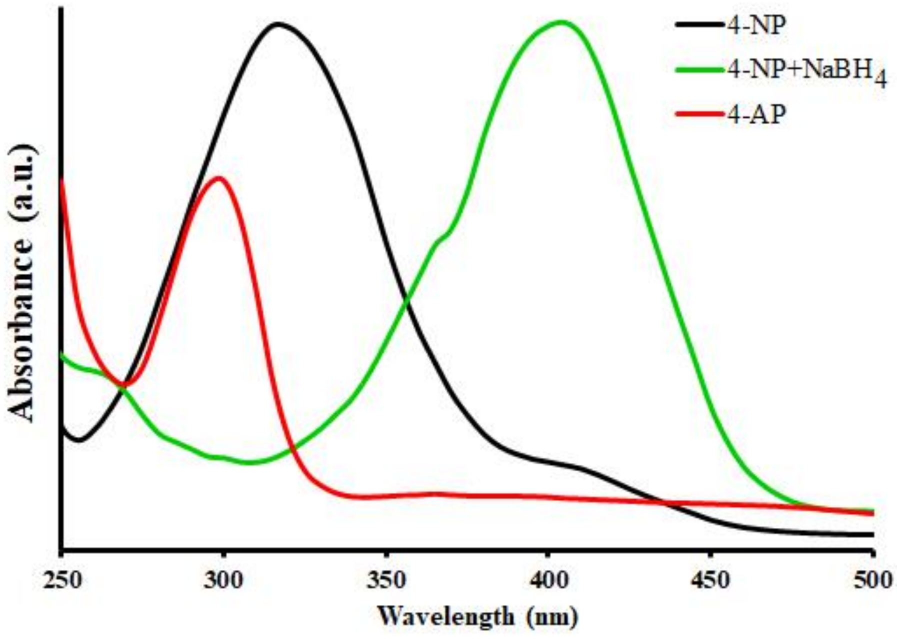

Figure S16. The UV-vis spectra of 4-NP, 4-NP after adding fresh solution of $NaBH_4$ and 4-AP.

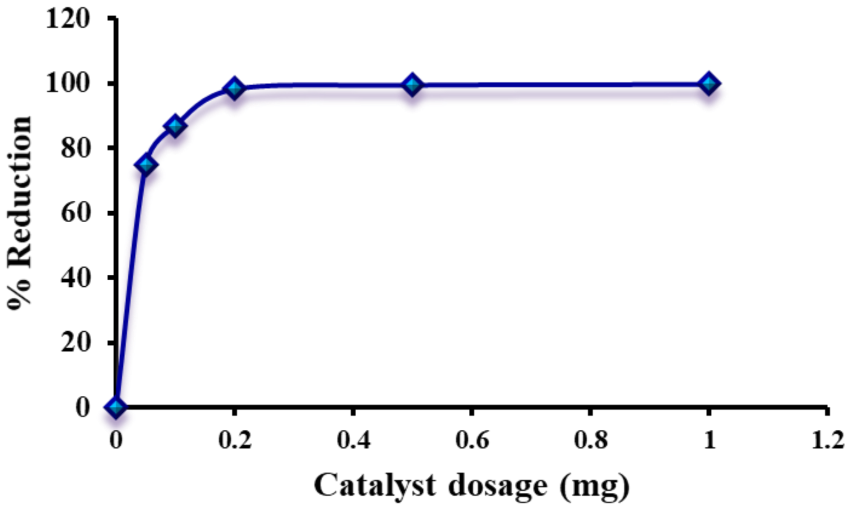

Figure S17. The 4-NP reduction performance in presence of various amounts of QH-240 catalyst.

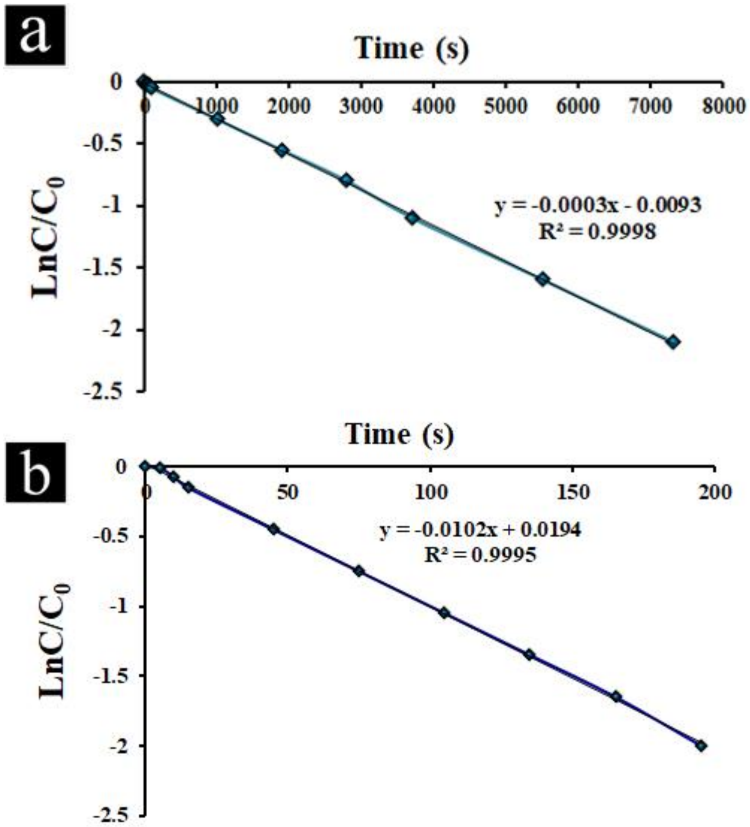

Figure S18. Plots of $\ln(C/C_0)$ versus reaction time for the reduction of 4-NP over a) HKUST and b) QH-240 catalysts.

Table S1. BET surface area, total pore volume, micro- and mesopore volume of the synthesized MOFs.

Sample	S_{BET} (m^2g^{-1})	V_t (cm^3g^{-1})	V_{micro} (cm^3g^{-1})	V_{meso} (cm^3g^{-1})
HKUST	1182	0.489	0.462	0.027
QH-200	913	0.436	0.369	0.067
QH-240	160	0.122	0.06	0.062
QH-260	5.6	0.046	0.002	0.044

Table S2. Hydrogen evolution while the hydrogenation of 4-NP to give selective 4-AP was taken place

Material	Time	H_2 evolution µmol · g without 4 NP	H_2 evolution µmol · g while working
HKUST-1	5h	9.54	4.50
QHKUST 240	5h	13.73	9.02

Table S3. Density of strength of acid sites in HKUST and QH-240 catalysts according to NH_3-TPD measurements.

Catalyst	weak sites		Medium sites		Total active acid sites ($mmolg^{-1}$)
	Peak position (°C)	Value ($mmolg^{-1}$)	Peak position (°C)	Value ($mmolg^{-1}$)	
**HKUST**	170	9.1	309	12.6	21.7
**QH-240**	172	1.7	313	46.8	48.5
